# Exploring the potential of using simulation games for engaging with sheep farmers about lameness recognition

**DOI:** 10.3389/fvets.2023.1079948

**Published:** 2023-02-23

**Authors:** Matt L. Jones, Maxwell S. Barnish, Robert R. Hughes, Aimee K. Murray, Omid Mansour, Tiziana Loni, Holly M. Vickery, Myfanwy L. Evans, Laura Green, Nervo Verdezoto

**Affiliations:** ^1^European Centre for the Environment and Human Health, University of Exeter Medical School, Knowledge Spa, Royal Cornwall Hospital, Truro, United Kingdom; ^2^University of Exeter Medical School, South Cloisters, University of Exeter, Exeter, United Kingdom; ^3^Faculty of Engineering, University of Bristol, Bristol, United Kingdom; ^4^School of Computer Science and Informatics, Human-Centered Computing Group, Cardiff University, Cardiff, United Kingdom; ^5^Independent Researcher, Dundee, United Kingdom; ^6^School of Agriculture, Policy and Development, University of Reading, Reading, United Kingdom; ^7^Independent Researcher, Dolgellau, United Kingdom; ^8^Life and Environmental Sciences, University of Birmingham, Birmingham, United Kingdom

**Keywords:** lameness, games, human-centered design, sheep, livestock, agriculture, farming, stockmanship

## Abstract

**Introduction:**

Computer simulation games are increasingly being used in agriculture as a promising tool to study, support and influence real-life farming practices. We explored the potential of using simulation games to engage with sheep farmers on the ongoing challenge of reducing lameness. Working with UK stakeholders, we developed a game in which players are challenged with identifying all the lame sheep in a simulated flock. Here, we evaluate the game's potential to act as a tool to help assess, train and understand farmers' ability to recognize the early signs of lameness.

**Methods:**

Participants in the UK were invited to play the game in an online study, sharing with us their in-game scores alongside information relating to their real-life farming experience, how they played the game, and feedback on the game. Mixed methods were used to analyze this information in order to evaluate the game. Quantitative analyses consisted of linear modeling to test for statistical relationships between participants' in-game recall (% of the total number of lame sheep that were marked as lame), and the additional information they provided. Qualitative analyses of participants' feedback on the game consisted of thematic analysis and a Likert Scale questionnaire to contextualize the quantitative results and identify additional insights from the study.

**Results:**

Quantitative analyses identified no relationships between participants' (*n* = 63) recall scores and their real life farming experience, or the lameness signs they looked for when playing the game. The only relationship identified was a relationship between participants' recall score and time spent playing the game. Qualitative analyses identified that participants did not find the game sufficiently realistic or engaging, though several enjoyed playing it and saw potential for future development. Qualitative analyses also identified several interesting and less-expected insights about real-life lameness recognition practices that participants shared after playing the game.

**Discussion:**

Simulation games have potential as a tool in livestock husbandry education and research, but achieving the desired levels of realism and/or engagingness may be an obstacle to realizing this. Future research should explore this potential further, aided by larger budgets and closer collaboration with farmers, stockpeople, and veterinarians.

## Background

Lameness is a change in animal gait that has various underlying causes, but is typically caused by bacterial infections of the hoof and foot (especially scald and foot rot) in farmed sheep, goats and cattle (1). As a macro-level manifestation of microbial ailments, the first diagnosis of lameness can typically be made by farmers after visual observation of their livestock walking. Despite this, lameness is still a major burden on livestock farming, with some evidence that this is partly because farmers differ in their ability to recognize lameness, especially in its early stages (2, 3). In UK sheep farming, lameness is estimated to cost farmers between £3.90 and £6.30 per ewe per year (4), and the industry as a whole £28–80 million per year (5, 6). As well as economic costs associated with veterinary expenses and livestock productivity losses, lameness also constitutes a substantial animal welfare (7, 8) and antibiotic stewardship problem (9), making it a priority issue for the sheep farming industry to address. In 2011, the Farm Animal Welfare Council (FAWC) challenged UK sheep farmers to reduce the average prevalence of lameness on UK sheep farms to less than 5% by 2016 and less than 2% by 2021—targets that were, at the time, considered achievable using evidence-based techniques (7). Whilst the initial 5% target appears to have been met—with a well-randomized study estimating the mean flock prevalence of lameness in the UK to be 3.5% (ewes) in 2013 (10)—there are signs that progress may have since stalled. The most recent (though non-randomized) study estimated a mean flock prevalence of lameness (ewes) of 3.2% in the 2018–2019 period, suggesting that farmers were not on track to reach the 2021 2% target (11). Furthermore, there are indications of limited uptake and farmer skepticism toward some of the lameness-reduction techniques recommended by the FAWC (11, 12), and that the numbers of farmers practicing key effective treatments may be reducing over time (13). Collectively, these observations suggest that new approaches might be needed to facilitate knowledge exchange between farmers and other interested parties to reduce lameness in the UK.

One new strategy to facilitate knowledge exchange between farmers and non-farmers that has recently been explored in agricultural education and research is the use of game-based approaches to facilitate innovation, participation, and multiple stakeholders perspectives (14, 15). The progress of information and communication technology (ICT) has led to the development of farm-based computer and video games worldwide that have actively engaged players in virtual farming environments (16). Indeed, computer-mediated virtual agricultural environments are well-established as mass-appeal simulation video games such as FarmVille and Farming Simulator, which serve as forms of entertainment for non-farmers and farmers alike (17). However, more recently, virtual environments have begun to be used as pedagogic and research tools for engaging with farmers in order to address serious, real-world issues. Most commonly, researchers have explored the use of virtual environments for educational purposes, having benefits such as making agricultural training more logistically feasible, affordable and accessible (18). Several projects have developed and explored the potential of games of this sort—including developing games for teaching crop cultivation and livestock breeding skills (19, 20), developing more all-encompassing agricultural training games (21, 22), and exploring the potential of virtual reality-assisted agricultural training (18). Virtual agricultural environments may also serve less obvious knowledge exchange purposes; for example, to encourage the adoption of precision agriculture technologies (23); to exchange knowledge and perspectives on farm design among farmers, researchers and advisors (24); to facilitate information sharing among farmers and with non-farmer stakeholders dealing with agricultural issues (15, 25). The idea of using virtual environments as tools for engaging with farmers is thus being taken increasingly seriously; representing a new, innovative, participatory, and even fun approach to understanding and addressing the real-world challenges of modern agriculture.

Here, we explore the potential of using computer-based gaming as an innovative approach to engage with UK sheep farmers and other stakeholders on the issue of the early recognition of the signs of lameness. Sheep lameness can be graded according to increasing severity of change in gait, and sheep farmers recognize different severities of lameness innately (26). Farmers that report that they recognize, catch and treat the first mildly lame sheep in a group experience lower prevalences of lameness compared to farmers who wait until sheep are more severely lame before they catch them (10, 27). Following a human-centered design approach, we developed a game (The Lameness Game) that is intended to support lameness reduction by serving as a tool to help assess, train and understand farmers' ability to recognize the early signs of lameness. We evaluated our game through an online evaluation study with participants playing and giving expert feedback on our prototype game, reporting our analysis of their in-game performance and feedback in order to assess the games' potential.

## Materials and methods

### Description of the lameness game

**Figure 1:**
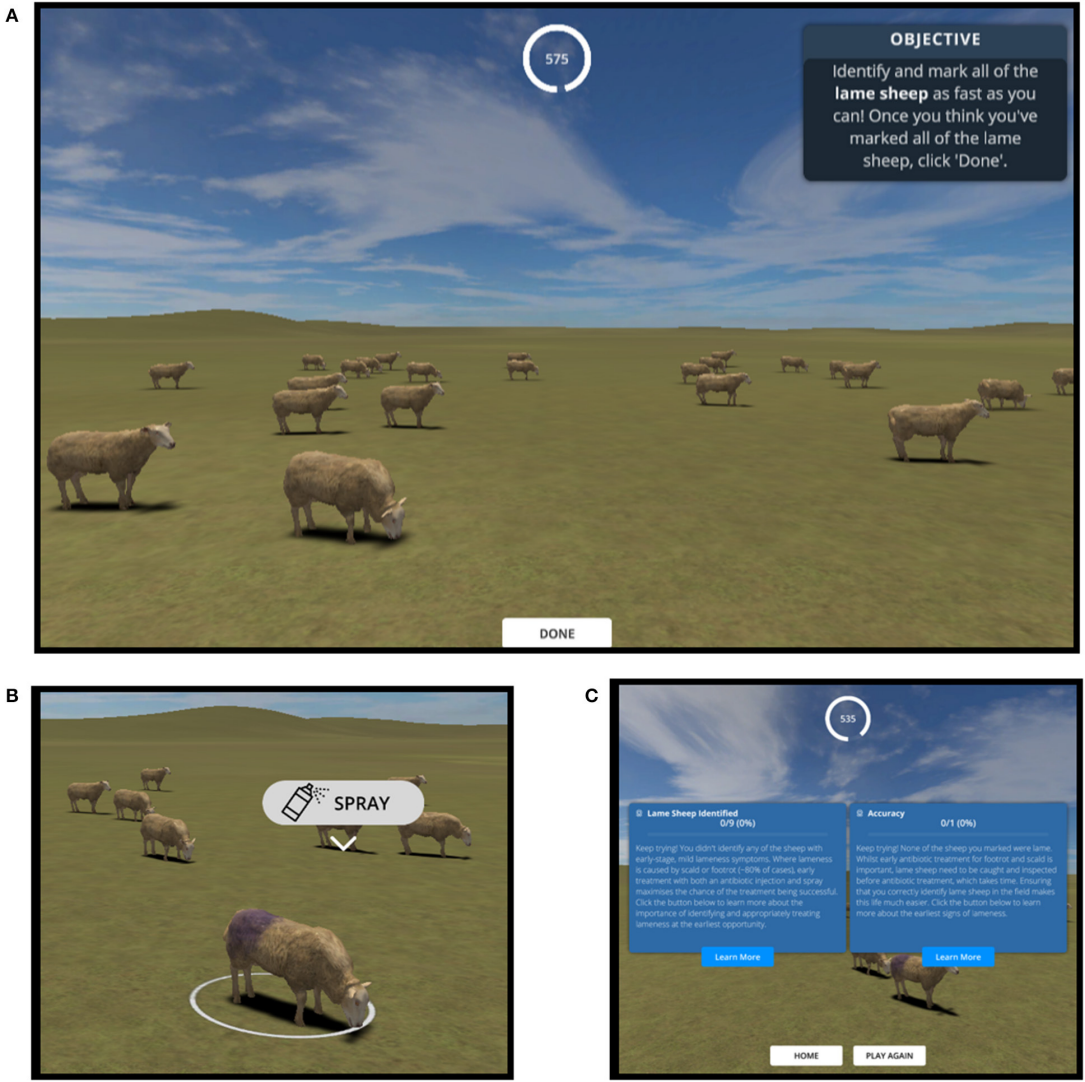
Screenshots of the game summarizing its main features. **(A)** In the game, players are presented with a field of virtual sheep and the goal of observing them to identify those with a lame gait. **(B)** Users can zoom in and select sheep, spraying them purple to mark them as lame. **(C)** At the end of the game (10 min timer ends or users click “Done”), users are presented with scores based on how many of the sheep they marked as lame were actually lame, as well as some related educational information.

Our game was a single-player, casual simulation game in which players were set the goal of identifying all of the lame sheep in a virtual flock in the shortest time possible (Figure 1). During gameplay, the displayed environment resembles a farm field which is occupied by virtual sheep programmed to spend most their time grazing (~73% of the time) or standing (~23.5% of the time), but that occasionally walked (~3.5% of the time). These parameters were intended to be somewhat reflective of estimated real-life ovine activity budgets whereby walking constitutes a minority (~2–4%) of the total activity (1, 28), whilst also providing a small (but not impractically small) window of opportunity to identify lame sheep within the time-frame of a relatively short game. Players could navigate the environment with game controls that resemble those of a simplified real-time strategy game; up-down-left-right to move the camera to move the camera across the field (WASD keyboard keys), camera rotate to change the direction of camera (Q & R keyboard keys) and zoom controls to change the field of view of the camera (trackpad/mouse scroll). At the start of the game, a “healthy” or “lame” status is randomly assigned to each of the 24 sheep in the flock (i.e., on average 50% of the sheep were assigned to be lame *via* a coin-flip style mechanism, though this was not disclosed to the player), which determines the animation used when they walk (Figure 1A). In our game, lame sheep exhibited a shortened stride on one (infected) leg, a quickened stride on the opposite leg, and a slight nodding of the head (Supplementary Video 1)—approximating the signs of early lameness represented by Score 2 on the Kaler et al. (29) scale. When players identified a sheep they thought was lame, they could select it by clicking it with the left mouse button, upon which an icon appeared above the sheep's body that the users could click to mark the sheep as lame (Figure 1B). The sheep was then marked with a purple spray and its status changed to “Marked as Lame” for the purposes of the in-game scoring system. At the end of the game, users received a score for their accuracy (% of sheep marked that were actually lame) and recall (% of the total number of lame sheep that were marked as lame), some educational feedback on their performance, as well as the time remaining on the in-game clock (Figure 1C). Players were given a maximum of 10 minutes to identify the lame sheep, but could choose to terminate the game and get their results early by clicking “Done.”

The game was developed using a human-centered design (HCD) process in which potential users (farmers, farm veterinarians and academics in the field) were involved throughout all stages of the design process (30), and substantially shaped the final game we evaluate here (Supplementary material 1; Supplementary Figure 1). The final game was built using game-programming software Unity (31) and 3D modeling software Blender (32) in collaboration by a game programmer (OM) and 3D artist/animator (TL) using a mix of pre-made, modified and newly-created 3D models, animations and other digital assets (33–37). The game runs standalone in a browser on desktop and laptops. At the time of publication, the online version of the game used in the study remains available (https://wheres-woolly.itch.io/lameness-game), and can still be played free of charge (preferably using the Google Chrome browser). An offline version of the game is also archived at Zenodo (https://doi.org/10.5281/zenodo.7612059), and can be downloaded and played locally.

### Evaluation of the lameness game

The game was evaluated *via* a 6-week online study (May–June 2021) in which those with and without agricultural experience were invited to play the game online and fill in an after-game questionnaire *via* the Microsoft Forms platform (Supplementary material 2). Through the after-game questionnaire, participants shared with us their in-game scores (those presented *via* the screen shown in Figure 1C) alongside feedback on the game. Participants were enrolled in the study by advertising it on social media and private mailing lists (targeting groups of interest where possible e.g., sheep societies), as well as during a workshop with University of Bristol Farm Animal Discussion Group (comprising veterinary practitioners, teaching staff and researchers). Participation was incentivized by offering participants entry into a lottery to win one of three £50 vouchers for an online farm supplies shop in return for the ~30 min of participation time. This study was approved by the College of Medicine and Health research ethics committee at the University of Exeter (application number 21/01/275). To comply with ethical requirements, participants were required to read an information sheet and digitally sign a consent form before participating in the study.

#### Participant recall scores in the game

Quantitative evaluation of the game consisted of analyzing the relationship between participants' recall scores in the game and data relating to their real-life experience and how they played the game (all self-reported in the after-game questionnaire; Supplementary material 2). Our logic was that the game could serve as a tool for training, testing or studying real-life lameness recognition practices if participants were able to translate real-life experience and skills into higher in-game recall scores. Recall was calculated and reported alongside accuracy at the end of the game (Figure 1C) and as for all other data, participants shared these scores with the research team *via* the after-game questionnaire.

In order to test whether participants had played the game as intended before engaging in further analysis, we first used D'agostino's test to test for normality and skewness in participants' recall and accuracy scores. A range of recall scores is expected to be underpinned by generally high (negatively skewed) accuracy scores (i.e., the majority of scores above >50%) if participants had successfully engaged with the goal of the game (to mark all the sheep they think are lame) without “cheating” (i.e., by taking a “shotgun” approach and marking all sheep as lame). High accuracy scores also gave us a first indication that our animations of lameness were at least realistic enough for participants' to recognize as lameness.

Subject to confirming this, we then proceeded with a more quantitative analysis of participants' recall scores; seeking to identify a feasible linear model describing what (if anything) affected participants' recall scores (subject to them meeting the assumption of normality). In order to do this, a *post-hoc* power analysis was first performed to understand how complex a model we could build with the sample size (power) available. Accounting for our sample size (*n* = 63), assuming stringent 95% power and 5% significance thresholds, and the use of a linear model with 1 on 61 degrees of freedom (i.e., a single continuous or two-factor explanatory e.g., true-false type variable), we estimated that our study had the power to detect an approximately “medium-to-large sized” effect (*f*^2^ = 0.21), *sensu* Cohen (38). Accordingly, we tested different candidate linear models—each with a single explanatory variable describing what drove participants' ability to identify lame sheep in the game—until a feasible model was identified. Beginning with our first hypothesis that there was a relationship between participants' in-game scores and their real-life farming experience (“Farming Experience” hypothesis), we progressed through to models testing for an effect of lameness signs participants looked for during the game (“Lameness signs looked for” hypothesis), and finally for an effect of more idiosyncratic factors to do with user engagement (“User engagement” hypothesis). To choose the explanatory variable computed in each model considered, we used an exploratory data analysis approach (39); plotting all variables relating to the hypothesis under consideration, and then choosing the variable(s) that visually appeared to have the strongest effect on recall scores for modeling (helping to mitigate against issues caused by multiple hypothesis testing). For the “Farming Experience” hypothesis, candidate variables plotted and chosen from where: whether or not the participant had experience in farming/related field (TRUE/FALSE categorical variable of two levels derived from Q15 in the questionnaire); the perceived annual prevalence of lameness they had experienced if they had farming experience (categorical variable of two levels derived from Q19 in the questionnaire); the number of years they had spent working with sheep if they had farming experience (continuous variable derived from Q19 in the questionnaire). For the “Lameness signs looked for” hypothesis, the candidate variables were the nine signs of lameness that participants told us they did or did not look for e.g., uneven posture, shortened stride on one leg when walking (TRUE/FALSE categorical variables of two levels derived from Q13 in the questionnaire). For the “User engagement” hypothesis the candidate variables were: how many times the participant had played the game before submitting their scores (categorical variable of five levels derived from Q5 in the questionnaire); whether or not the participant had problems with the game's controls (TRUE/FALSE categorical variables of two levels derived from Q7 in the questionnaire); observing type/how the participant observed the sheep when playing the game (categorical variable of three levels derived from Q10 in the questionnaire); moving type/how the participant moved around the flock when playing the game (categorical variable of four levels derived from Q11 in the questionnaire); whether or not the participant completed the pre-game tutorial (categorical variables of three levels derived from Q6 in the questionnaire); the computer set-up/pointing device the participant used (categorical variables of three levels derived from Q7 in the questionnaire); and the time spent playing the game (continuous variable derived from Q2 in the questionnaire). In total, we tested four models—one for the “Farming experience” hypothesis, two for the “Symptoms looked for” hypothesis, and one for the “User engagement” hypothesis. *P*-values from each of the models were Bonferroni-corrected according to the number of previous models tested, and we stopped building models once a feasible model was identified (i.e., one with a *p*-value < 0.05). Our null hypothesis (H0) in all models was that our measured variable(s) did not affect participants' recall, whilst our alternative hypotheses was that the variable under consideration affected participants' recall.

During this main analysis, one minor additional analysis was performed to test for differences in the lameness signs participants looked for according to real-life farming experience (whether or not the participant had worked in farming or a related field). This aided our understanding of the data and helped to justify investigating the effect of the lameness signs looked for on recall scores (by establishing whether this effect was likely to be already captured by the farming experience effect). This analysis consisted of a chi-squared test performed on a 9 × 2 contingency table of the number of participants that looked for each category of lameness signs (nine TRUE/FALSE categories), according to farming experience (two TRUE/FALSE categories).

All analyses were performed in the R programming language (40) implemented *via* RStudio (41). Exploratory plotting to identify candidate variables for linear modeling was conducted using base R functions and the *beeswarm* function of the “beeswarm” package (42). Given that accuracy and recall scores were percentage data, they were both arcsine square root transformed using base R functions before being subjected to statistical testing (D'agostino's test and linear modeling). D'agostino's test was implemented *via* the *agostino.test* function of the “moments” package (43). Power analysis was implemented *via* the *pwr.f2.test* function of the “pwr” package (44). Linear modeling and Bonferroni correction of *p*-values was performed using base R functions *lm* and *p.adjust*, respectively. For the additional analysis, the contingency table was visualized using the *balloonplot* function of the “gplots” R package (45) and the chi-squared test was performed using the base R function *chisq.test*.

#### Feedback on the game from those with real-life farming experience

To help explain the results from the quantitative analysis of participants' recall scores and evaluate the game more broadly, we also collected feedback on the game from participants who had real life farming experience and conducted complementary qualitative analyses. We limited this data collection and evaluation to participants who had worked in farming or a related field (i.e., those who had answered “Yes” to the question “Have you ever worked in farming or a related field e.g., farm vet?”) because this was the intended audience of the game. These participants with real-life farming experience directly evaluated the game in two ways; by providing open-form feedback in the after-game questionnaire, and by scoring evaluation statements on a Likert scale.

Open-form feedback provided an opportunity for participants to elaborate on their thoughts about the game and suggest new potential uses of it. This feedback was analyzed using inductive thematic analysis, a qualitative analytical technique that involves finding patterns in a non-numerical dataset to understand participants' opinions, perspectives and experiences (46, 47). Thematic analysis values all participants' perspectives without privileging the more commonly/frequently expressed perspectives that might prioritize the quantification of patterns e.g., coding reliability approaches, underpinned by positivist approaches and quantitative methods (47, 48). We conducted thematic analysis on free-text feedback from those who provided it. Statements were coded and then reported in terms of themes, each consisting of one or multiple conceptually linked sub-themes. Supporting quotes were noted to illustrate each sub-theme. Analysis was initially conducted independently by two researchers (MSB and NVD) reading and coding all free-text feedback and identifying the initial themes. Any discrepancies (e.g., disagreements in assignment of comments to themes, comments fitting more than one theme) were initially discussed between these two researchers then an agreed analysis was circulated to three further researchers (MLJ, RH, and AM) for peer validation, feedback and finalization.

In the Likert scale sub-questionnaire, participants rated the game on such factors as its educational, realism and entertainment value—potential uses of the game that we had in mind when designing it in consultation with stakeholders (Supplementary material 1). Since this data was only collected for one group (those with real-life farming experience), there was no formal analysis of this data and the data were only plotted and described to qualitatively inform the interpretation of results and evaluation of the game.

## Results

### Study participants

A total of 63 people participated in the study, after the removal of one participant who said that the game did not work properly for them. Thirty-two participants had not worked in farming or a related field, and 31 had worked in farming or a related field. Of those with farming experience, the majority (30/31) had worked with sheep either as farmers (12/31), stockpeople (8/31), veterinarians (9/31), or in other roles (9/31) such as livestock technicians or in agricultural research or policy (N.B. individual participants sometimes had experience in multiple fields, hence numbers do not total 31). Most of those who shared information about the levels of lameness they had experienced in the flocks with which they had worked said that they had experienced annual lameness levels of between 5 and 10% (13/29).

### Participant recall scores in the game

**Figure 2:**
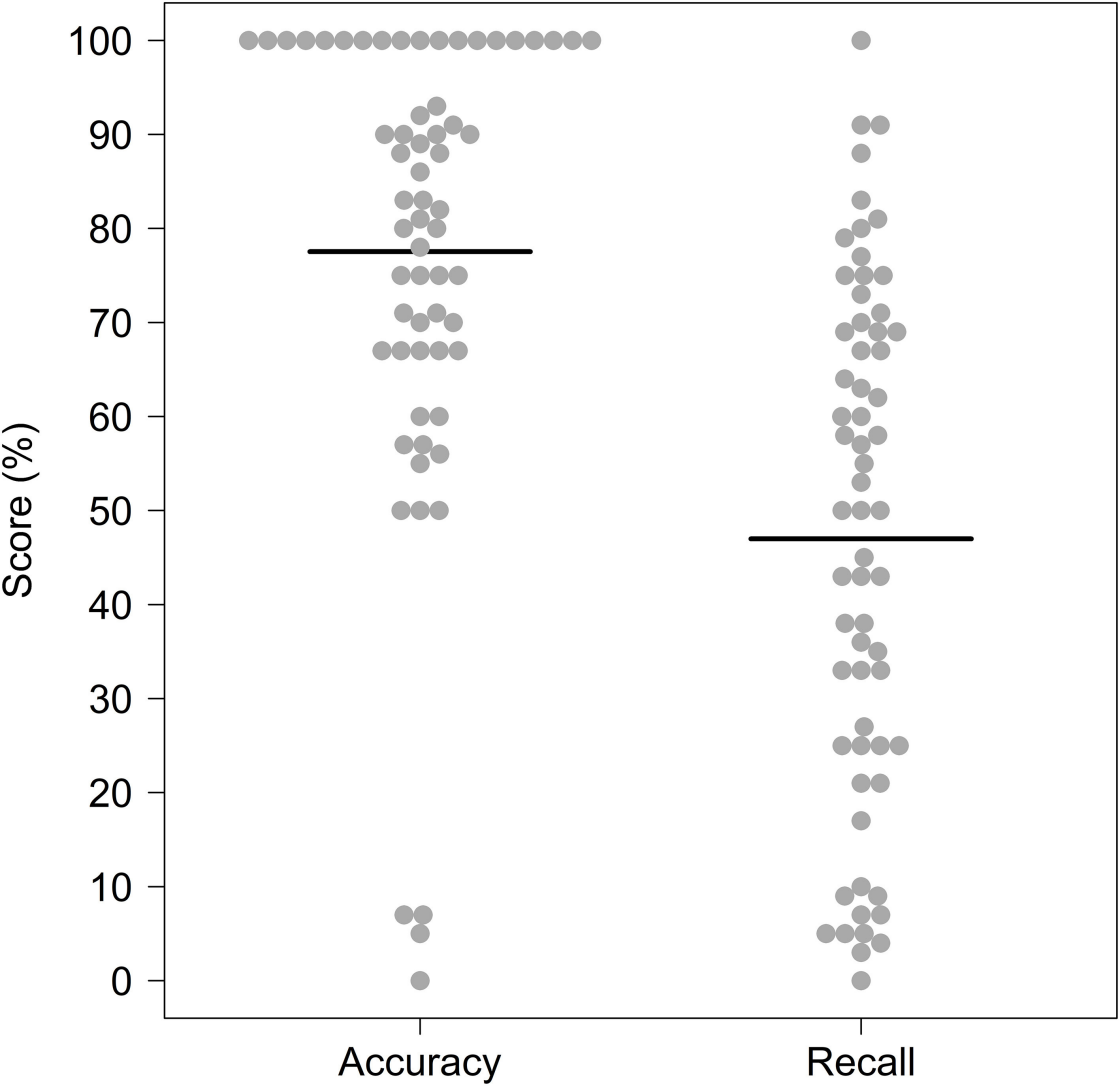
Comparison of distributions of participants' (*n* = 63) accuracy (i.e., number of sheep they marked as lame that were actually lame) and recall (i.e., number of the total lame sheep in the flock that they marked) scores. Individual participant data points are jittered using the beeswarm algorithm (R Package “beeswarm”) and mean recall scores are plotted as bold horizontal lines underneath the data points.

Participants' accuracy and recall scores were distributed as expected, permitting deeper analysis of participants' recall scores (Figure 2). The majority of participants (89%) had accuracy scores above 50% (D'Agostino's test; skew = −1.53, kurtosis = −4.24, *p* = < 0.01), indicating that they were not simply “cheating” the game by taking a “shotgun” strategy of marking all or most of the sheep as lame in order to maximize their recall scores. High overall accuracy scores also indicated that our animations of lameness were at least realistic enough for participants to recognize them as lameness, further indicating that variation in recall scores was likely to reflect some level of skill in spotting lameness. Recall scores themselves were normally distributed across the entire percentage range (Figure 2; D'Agostino's test; skew = −0.12, kurtosis = −0.44, *p* = 0.662), justifying a parametric analysis of the factors influencing participants' these scores.

#### Farming experience

**Figure 3:**
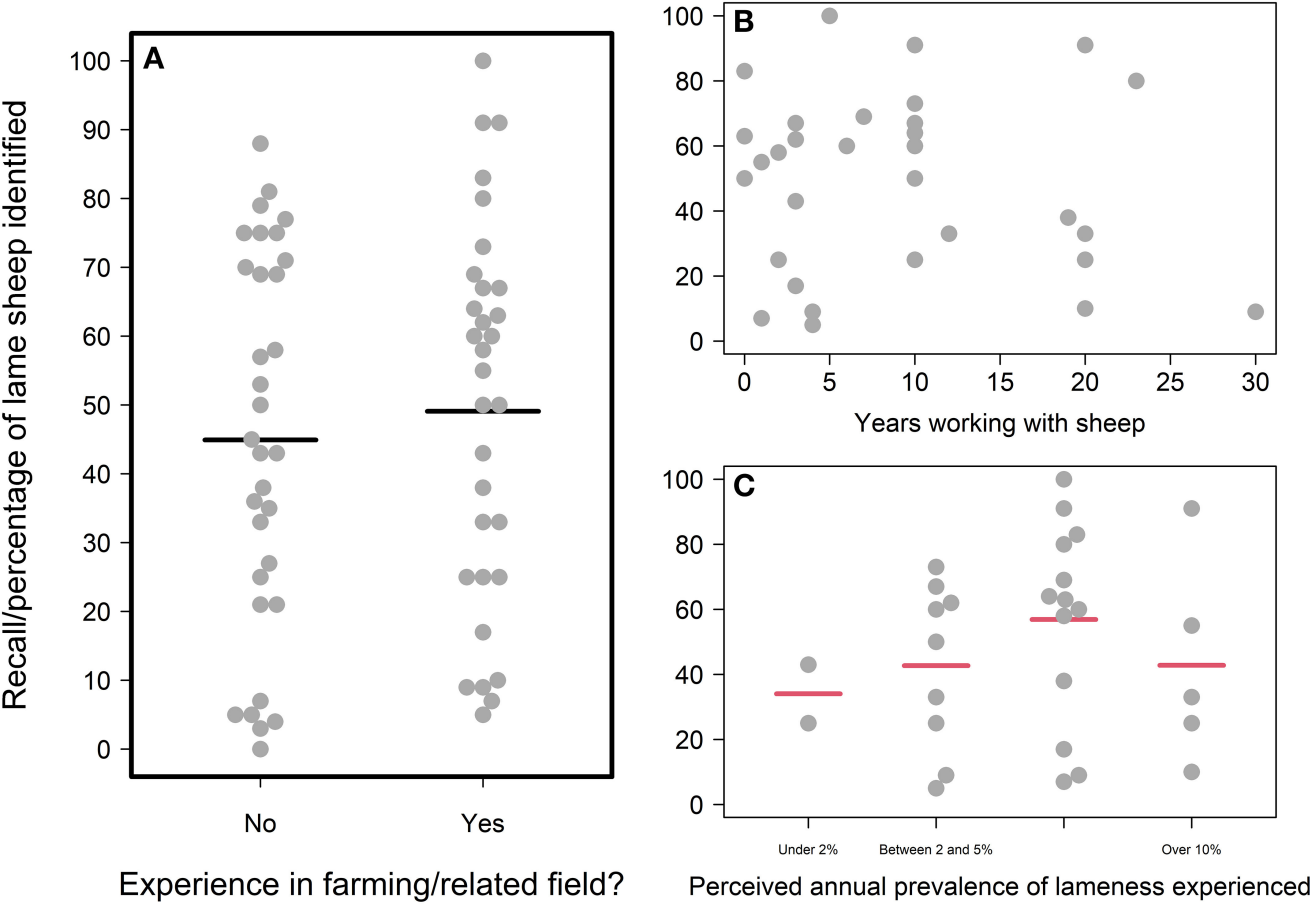
Relationships between participants' recall scores and their real-life farming experience. **(A)** Recall scores of those without and with farming experience; **(B)** Recall scores and years of farming experience spent working with sheep (for participants with farming experience). **(C)** Recall scores according to the perceived levels of lameness experienced in real-life flocks (for participants with farming experience who answered this question). For categorical variables, individual participant data points are jittered using the beeswarm algorithm (R Package “beeswarm”) and mean recall scores are plotted as bold horizontal lines underneath the data points. Mean recall scores colored red are those likely to be poor estimates due to small sample sizes i.e., the lower or upper quartile exceeds the 95% confidence limits of the mean. The plot is framed in a bold outline if that relationship was formally tested statistically.

There was no evidence that real-life farming experience was driving the variation in participants' recall scores (Figure 3). Initial visual examination of exploratory plots of the data identified no difference in recall scores according to whether participants had ever worked in farming or a related field (Figure 3A). We validated that this was not just because strong effects of sheep-specific farming experience were being obscured by noise from other types of farming experience; those who had worked with sheep as farmers, stockpeople, veterinarians, or in other roles had visually similar recall scores to those who had not (Supplementary Figure 2). Similarly, there were also no visually observable relationships between recall and the number of years the participants had spent working with sheep (Figure 3B), or the level of lameness those who had worked with sheep had experienced (Figure 3C)—again suggesting no higher-level relationships among those with farming experience. Formal statistical testing of the relationship between recall and whether or not the participant had worked in farming or a related field (which encompassed the entire dataset) revealed no significant difference. Those who had not worked in farming or a related field identified a similar percentage of the lame sheep in the game as those who had worked in such fields (“Farming Experience” model; $R_{\text{adj}}^{2}$ = 0.01, *p* = 0.425, *F* = 0.64, one on 61 DF; Supplementary Figure 3).

#### Lameness signs looked for

The lameness signs that participants looked for when playing the game were not differentiated by whether they had real-life farming experience (Supplementary Figure 4). Those with farming experience tended to more often look for lameness signs, but there was no statistical difference in the distribution of the signs they looked for compared to those without farming experience (*X*^2^ = 11.6, df = 8, *p* = 0.17). This suggested that there was potential for an effect of lameness signs looked for that was not already captured in the “Farming Experience” model.

**Figure 4:**
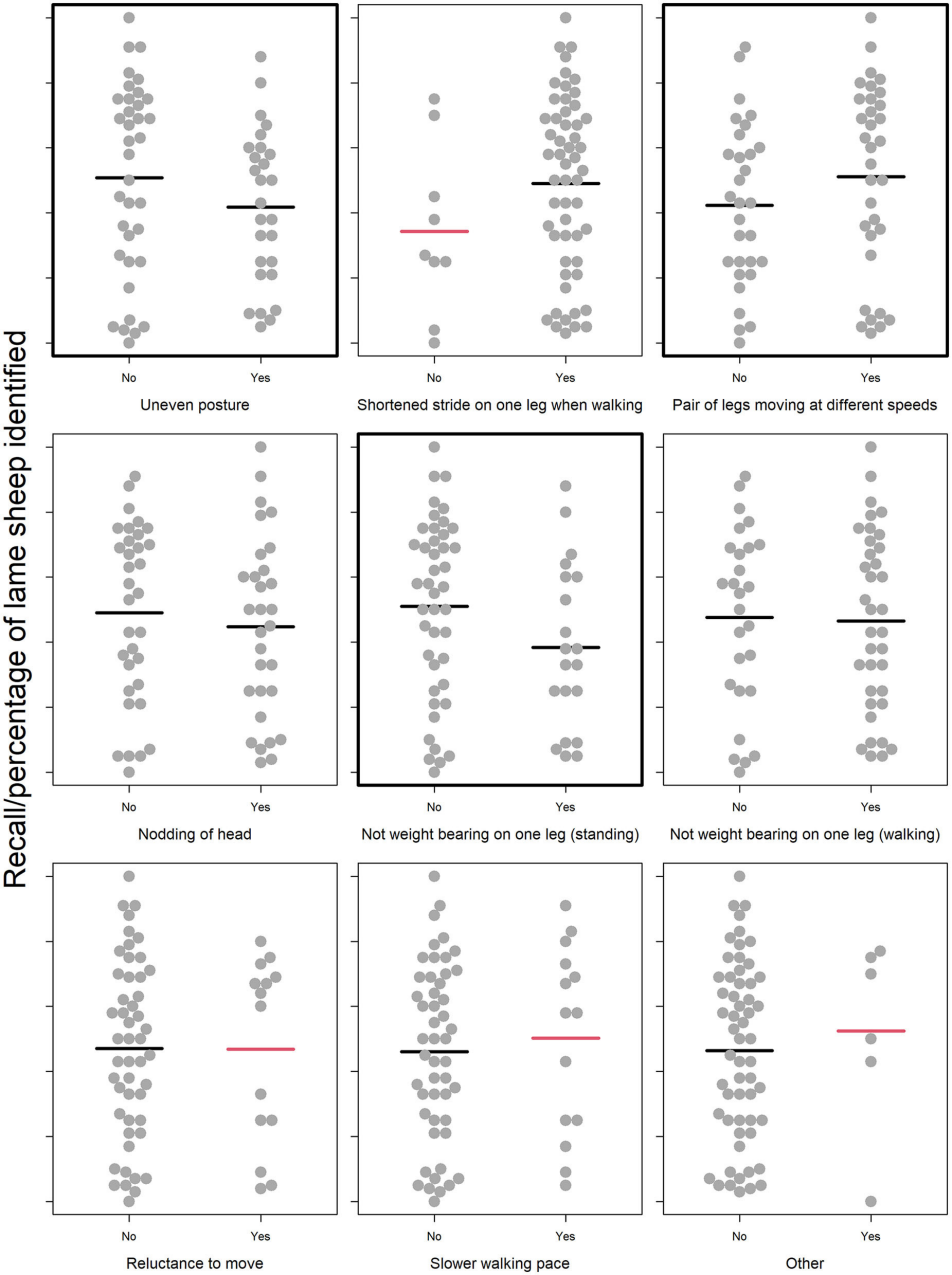
Relationship between participants' recall scores and the signs they looked for when playing the simulation game. Recall scores of participants that did not and did look for each of eight classic signs of various stages of lameness (29), plus an extra category of “Other” signs looked for which we asked participants to elaborate upon. For categorical variables, individual participant data points are jittered using the beeswarm algorithm (R Package “beeswarm”) and mean recall scores are plotted as bold horizontal lines underneath the data points. Mean recall scores colored red are those likely to be poor estimates due to small sample sizes i.e., the lower or upper quartile exceeds the 95% confidence limits of the mean. The plot is framed in a bold outline if that relationship was formally tested statistically.

However, when we explored this possibility using exploratory data analysis, no such effects were apparent. All of the relationships between in-game recall scores and the signs participants looked for were weak according to initial visual observation of the plotted data (Figure 4). Lameness signs that we included in the animation and deemed to be the most obvious signs of lameness in the game (uneven posture and nodding of the head) were not strongly related to participants' recall scores. For several signs, the number of participants looking or not looking for the sign was too small to accurately compare the two mean recall scores (red-colored mean lines). The three relationships with the strongest visual differences in the means were that participants who looked for uneven posture or differing leg speeds (i.e., a limp which we included in the animation as a more subtle lameness sign) scored higher, whilst those who looked for sheep unable to bear weight on a leg whilst standing (a sign of more advanced lameness that was not included in our animation of early lameness) scored lower. However, when statistically tested, neither looking for uneven posture (“Lameness signs looked for” model A; $R_{\text{adj}}^{2}$ = 0.02, *p* = 0.51, *F* = 1.32, one on 61 DF; Supplementary Figure 5), looking for a limp (“Lameness signs looked for” model A; $R_{\text{adj}}^{2}$ = 0.02, *p* = 0.666, *F* = 1.52, one on 61 DF; Supplementary Figure 6) or looking for a raised leg (“Lameness signs looked for” model B; $R_{\text{adj}}^{2}$ = 0.04, *p* = 0.458, *F* = 2.57, one on 61 DF; Supplementary Figure 7) were predictive of participants' recall scores.

#### User engagement

**Figure 5:**
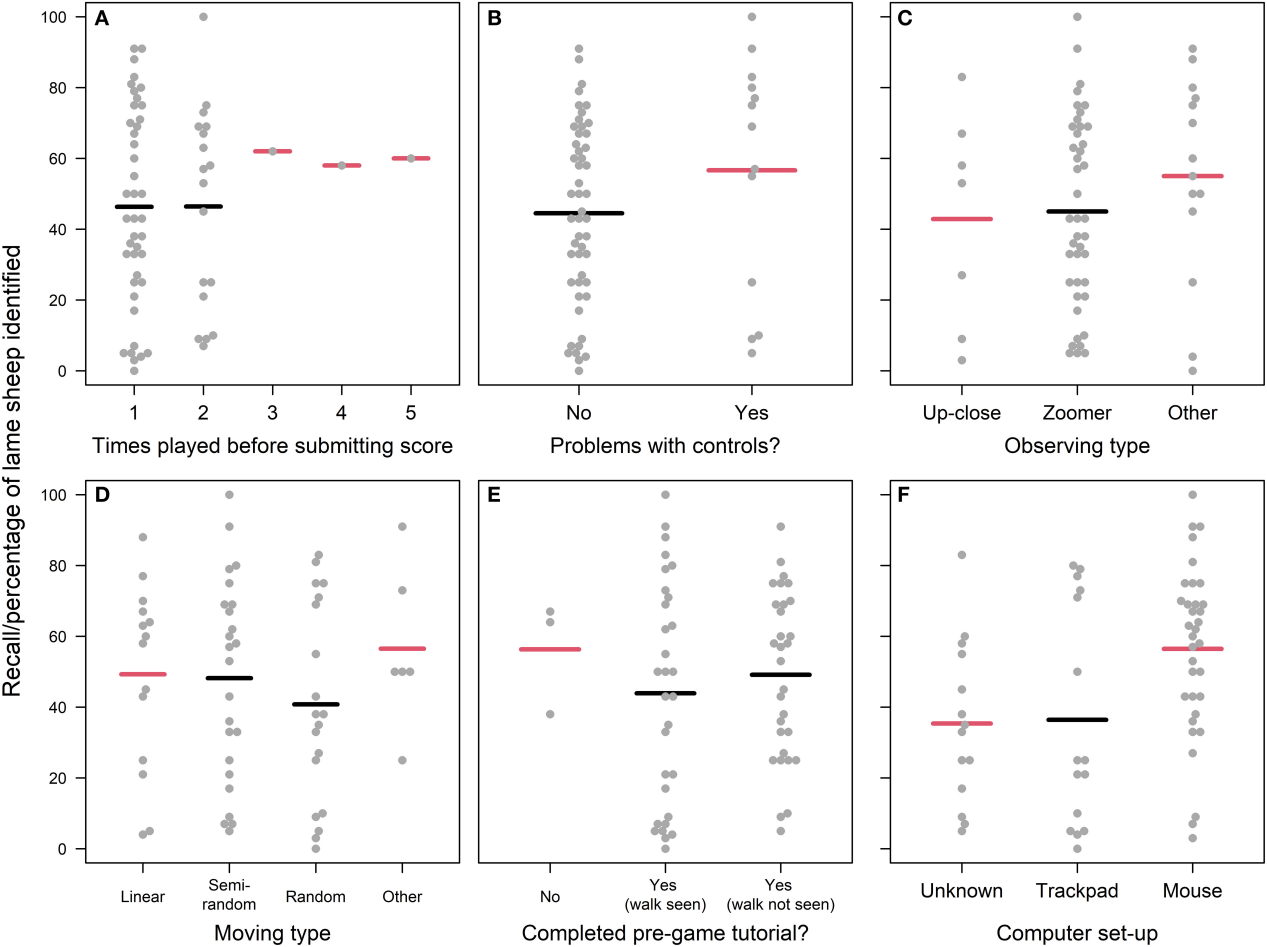
Recall scores of participants according to **(A)** how many times the participant played the game before submitting scores; **(B)** Whether the participant had problems using the game controls; **(C)** Whether the participant observed the sheep up-close, from afar and then zooming in, or using another strategy (e.g., combination of the two); **(D)** How the participant navigated the virtual field to identify sheep; **(E)** Whether the participant completed the pre-game tutorial; **(F)** The participant's computer set-up/pointing device. Individual participant data points are jittered using the beeswarm algorithm (R Package “beeswarm”) and mean recall scores are plotted as bold horizontal lines underneath the data points. Mean recall scores colored red are those likely to be poor estimates due to small sample sizes i.e., the lower or upper quartile exceeds the 95% confidence limits of the mean. The plot is framed in a bold outline if that relationship was formally tested statistically. For a more detailed explanation of what the categories mean (particularly for “observing type” and “moving type”) please refer to Supplementary material 2.

**Figure 6:**
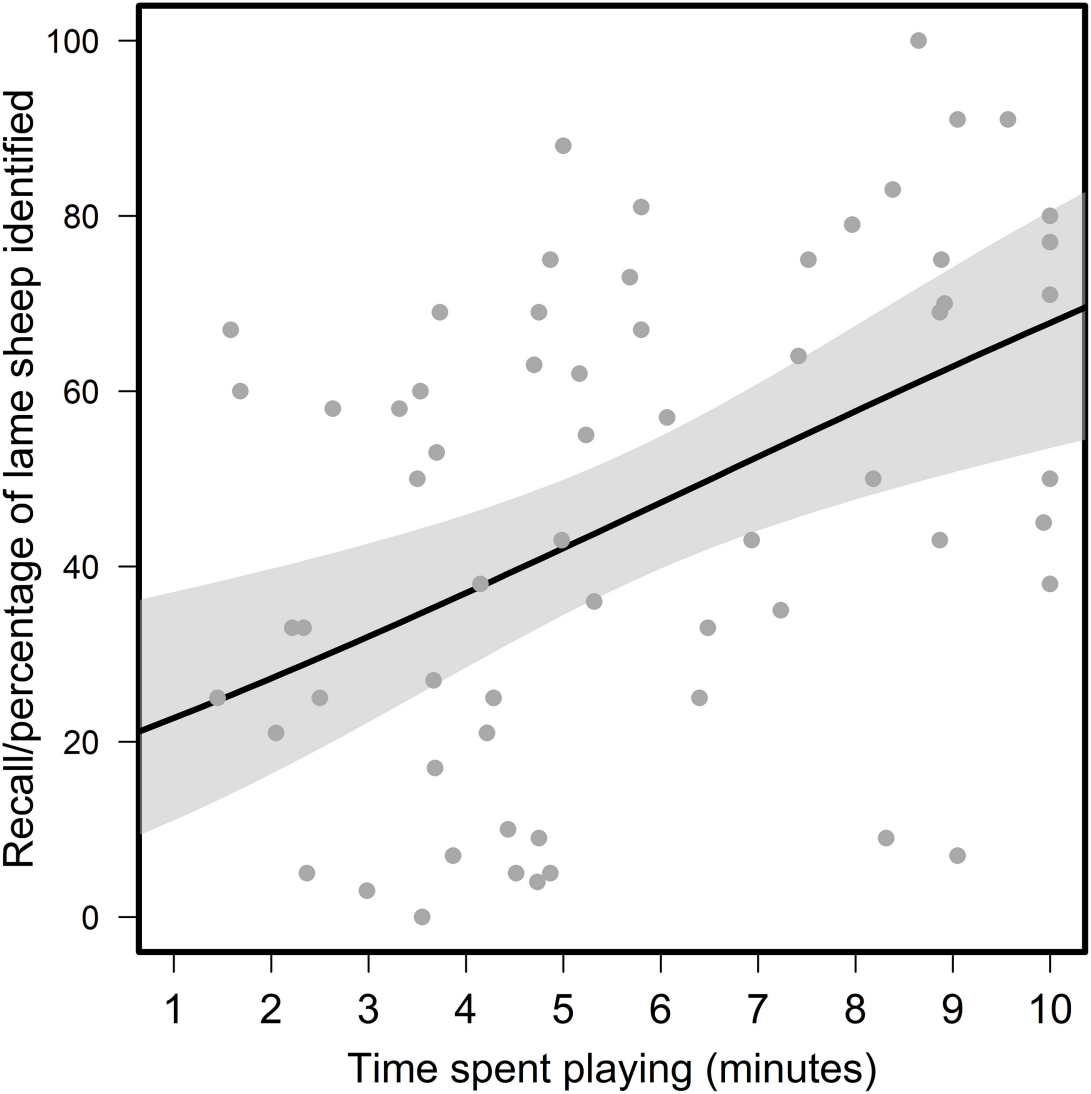
Relationship between participants' recall scores and the time they spent playing the game. Solid black line and shaded area represent the fit and 95% confidence interval of the linear model, respectively.

Similarly to the “Farming experience” and “Lameness signs looked for” variables considered, most aspects of participants' user engagement did not have a strong effect on recall scores, with recall scores either widely distributed within, or thinly spread across, the explanatory categories considered (Figure 5). The exception to this was that the time spent playing was positively and linearly related to in-game recall (Figure 6), which formal statistical testing confirmed (“User Engagement” model; $R_{\text{adj}}^{2}$ = 0.17, *p* < 0.01, *F* = 12.65, one on 61 DF; Supplementary Figure 8). Specifically, within the range playing lengths observed (1.45–10 min), participants identified an average of two additional sheep for every additional minute played.

### Feedback on the game from those with real-life farming experience

#### Feedback received as open-form responses

Nineteen out of 31 participants with real-life farming experience provided additional free-text feedback on the game and their experience playing it. During the qualitative thematic analysis (46) of these responses, five key themes emerged: the perceived realism of the game, reflective experiences, challenges of playing the simulation game, emotional responses to the game, and participants' suggestions for improvement Supplementary material 3.

##### Perceived realism of the game

Participants with real-life farming experience commented on their perceptions of how realistic the game was as a simulation of real-life experiences with sheep on the farm. Opinion regarding the realism of the simulation was split, with some participants considering that the simulation was “*really realistic*” and “*mimicked sheep well*,” and others expressing that they thought our animations were not sufficiently realistic to enable them to apply their real-life experience of spotting lameness in the game. For example, one participant simply remarked that the simulation was “*not realistic*,” while another noted in particular that “*the main issue was the unrealistic movement of the feet on the ground while standing*”—an animation bug that was known to researchers, but considered minor and impractical to fix before study initiation given timeframe/budget available.

##### Technical challenges playing the simulation game

Participants with real-life farming experience commented on a range of technical challenges relating to the game simulation that hindered their ability to engage with and benefit from the game. Four different aspects were identified as sub-themes: lack of movement of the sheep; simple, unnatural and confusing game simulation of sheep behavior; inability to mark non-lame sheep; usability and animation/simulation issues.

The first sub-theme, the lack of movement of the sheep, concerned the perceived staticness of the digital sheep and the inability of the player to affect it. Additionally, we considered that the challenge of spotting very subtle signs of lameness efficiently when only presented with glimpses of the behavior was a key skill to early identification of lameness in the flock. However, as one participant observed, “*lameness is not often identified when animals are static in the field, more often when animals are being moved or handled*.” A key issue for participants appeared to be that we did not fully simulate the real-life behavior of farmers “*working the flock*,” whereby the farmer or stockperson moves around and through the flock to stimulate sheep movement: “*I think most farmers would say that they also assess lameness by making the sheep walk / move away from them rather than just wait until they walk*.”

The second sub-theme, the “simple, unnatural, and confusing game simulation,” concerned distractions brought about by the games' computational performance as a consequence of the perceived realism of the game previously described. Commenting on the “foot slide” bug, one participant noted that while “*the sheep animations are good, but to a trained eye I found them confusing, e.g., none of them stood grazing in a normal posture because they were all jiggling their legs all the time*.” In addition to the “foot slide” bug, there were other technical challenges such as game lag and stilted movement, reflecting limitations of the technical systems involved in presenting the game to players online. For example, one participant commented that it was “*sometimes difficult to tell if a normal movement of sheep was a game lag*,” while another considered that the “*movement [was] stilted which made identifying slightly lame sheep virtually impossible*.”

The third sub-theme was the inability to mark non-lame sheep. The fact that there was no means to mark non-lame sheep in the game made it more difficult for participants to remember which sheep they had already assessed, though this was also an intentional design choice. We omitted this feature after discussion with our advisory board, because we considered that in real-life situations of assessing lameness, only lame sheep are usually marked. One participant's comment composed this theme, mirroring the difficult compromise between playability and realism that we encountered when designing the game: “*It was a bit frustrating not to be able to mark non-lame sheep when surveying, but that is more realistic and requires strategy*.”

The last sub-theme concerned usability and animation/simulation issues. A lack of smoothness in game animations was commented on by one participant in particular, who noted that this issue made “*the distinction between a normal walking gait and a limp less easy to discern*.” Meanwhile, another participant noted a lack of clarity in the graphics, which meant “*it was hard to see if they were holding a leg slightly up*.” Another participant also mentioned the “foot slide” bug, which was commonly commented on by participants from a range of perspectives, as reflected in the previous sub-themes.

##### Emotional responses to the game

Participants with real-life farming experience frequently used the open-form feedback request to express how they felt playing the game, with the 4 key sub-themes emerging in thematic analysis: enjoyment, interest, boredom, and frustration.

Some participants express positive feelings about the game such as enjoyment (sub-theme 1), saying that they “*enjoyed the game*” and found it “*entertaining*.” Others expressed interest in the game (sub-theme 2), with one commenting that it was “*interesting to be looking for signs in virtual sheep*” and another that they “*thought this was brilliant*.”

However, some participants also expressed negative feelings toward playing the game. Boredom (sub-theme 3) and frustration (sub-theme 4) were expressed, and appeared to be mostly related to the staticness of the sheep and their inability to affect it (theme 3: sub-theme 1). For example, one participant noted that they became “*bored waiting for the sheep to move*,” and similarly others commented that the game was “*frustrating*” or “*very frustrating*” to play (sub-theme 4), with one noting explicitly that the cause of their frustration was “*waiting for the sheep to move*.”

##### Reflective experiences

Participants with real-life farming experience also reflected on the experience of playing the game and the strategies they employed to identify lame sheep. For example, one participant emphasized how the game “*allowed me to get a better sense of my knowledge and skills*,” reinforcing how the game could enable participants to take stock of their current stockpersonship skills, and serve as a useful benchmarking exercise. However, others found the game too easy as one participant commented that “*lame sheep aren't always that easy to spot in a field*,” while another commented that “I *think most sheep farmers know the signs of lameness*.” Considering strategies, participants mentioned that in real life, it was important to “*walk around the flock*,” and noted that the sheep “*would move*” in response to the farmer's movements in a more realistic setting.

##### Participants' suggestions for improvement

Participants with real-life farming experience also offered suggestions for improvement to the game or to inform future games in this field. These suggestions fell into two broad categories.

Firstly, in line with other feedback, there were suggestions relating to making sheep move, e.g., using additional mechanisms and characters. Creating more natural movement patterns, rather than just a realistic gait, was considered an important priority for future improvement. Participants offered a range of perspectives on how to make the sheep move, but a common view was that it was important to be able to actively move the sheep, as a farmer would in a real-life field, rather than passively waiting for the sheep to move in order to be able to assess gait, as in the current game. For example, one participant suggested: “*If there was a way to make each sheep move, that would really help to keep engagement*.” Meanwhile, another participant suggested adding a sheep dog character to “*run round*” the sheep, while another suggested “*walking a person around so they [the sheep] walk away from you*.” It was commonly agreed that active flock management would be needed for the game experience to be realistic.

Secondly, other participants suggested providing additional visual or sound feedback in the game. One participant commented that visual feedback could be reinforced by offering a “*slightly more realistic depiction of sheep movement for non-lame sheep*,” while another participant considered that auditory feedback regarding the correct identification of a lame sheep, “*maybe a sound…as you chose the correct animals*,” could be a useful addition.

#### Feedback received *via* the Likert scale questionnaire

**Figure 7:**
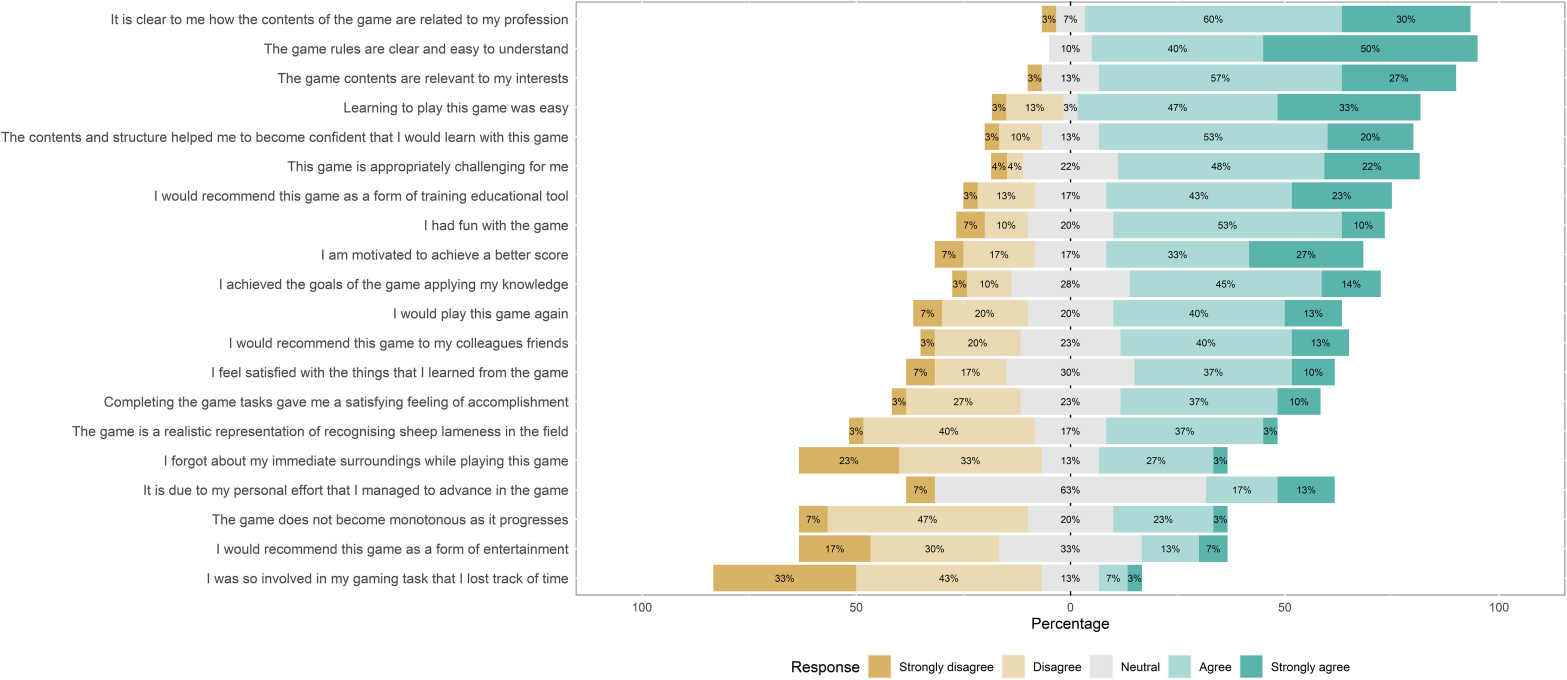
Quantitative feedback given on the game *via* a Likert Scale questionnaire. Statement rated are shown on the rows, with the total percentages of participants with farming experience responding negatively, neutrally and positively to the statements overlaid on the stacked bar graph.

30 out of 31 participants with real-life farming experience answered the Likert scale sub-questionnaire providing additional feedback on the game. Feedback suggested these participants could see the potential of games like ours as professional training-type tools in agriculture, but were unsure whether our prototype had realized this potential fully (Figure 7). The majority of participants agreed with statements related to the purpose (“It is clear to me how the contents of the game are related to my profession;” 90%) and usability (“The game rules are easy to understand;” 90%) of the game. Similarly, statements expressing the educational potential of the game—“Learning to play this game was easy” (80%), “The contents and structure helped me to become confident that I would learn with this game” (73%), and “I would recommend this game as a form of training/educational tool” (66%)—received agreement from the majority of participants. However, there was lower agreement with the statement expressing that this educational potential had been achieved (“I feel satisfied with the things that I learned from the game;” 47%). Regarding statements related to the realism of the game, there moderate agreement with the statement “I achieved the goals of the game by applying knowledge” (59%) and low agreement with the statement “The game is a realistic representation of recognizing sheep lameness in the field” (40%). Statements related to the entertainment value of the game received varied responses. Most participants felt the game offered an appropriate level of challenge (70%), and expressed that they had some fun playing (63%). However, many participants appeared to find the game boring by the end of playing; expressing that they felt the game became monotonous as it progressed (54%) and not recommending it as a form of entertainment (47%). The game was not deemed particularly absorbing, as reflected by the fact that most participants did not lose track of time (76%) or forget about their immediate surroundings (56%) while playing the game.

## Discussion

Our online evaluation study highlighted the challenges and opportunities of using simulation games for the purposes of supporting real-life livestock husbandry practices. Whilst positive feedback from participants indicated signs of potential for using simulation video games in this context, barriers to this audiences' user engagement with computer games like ours hindered this potential from manifesting more widely. Particular barriers included participants' apparent desire for high levels of realism and engagingness in the game—expectations which we struggled to meet and therefore limited the game's ability to function as a tool for quantitatively assessing, train and understand farmers' ability to recognize the earliest, subtlest signs of lameness. Nonetheless, the results of the study provide valuable insights for the design and use of future similar games and studies in livestock husbandry.

### User engagement shapes in-game performance where participants struggle to relate to the simulated environment

Somewhat unexpectedly, we could not detect any relationship between participants' recall scores and their real-life farming experience or the lameness signs they looked for when playing the game. Given the substantial variation in participants' recall scores, one possibility is that such a relationship existed, but was outweighed by the effects of unknown causes of variation that we did not measure *via* the after-game questionnaire. Another possibility is that the ability to detect lameness, whether real or simulated, is not related to expertise—as was previously suggested by a similar study of equine lameness recognition (49). Including a parallel test of participants' ability to identify real-life lameness (e.g., in a real flock or videos of one) in the study would have helped to clarify this (though was not practical to implement within the time-frame and resources of the current project). However, given the finding that the time spent playing was the only driver of participants' in-game performance, alongside the results of our qualitative analyses, we believe that the lack of an effect of livestock husbandry experience/skills was more likely to be the result of participants not finding the game sufficiently realistic or engaging.

Regarding realism, although participants' explicit statements about the game's realism were split, many of the other themes identified in participants' feedback related back, in some way, to the game not sufficiently reflecting real life. Statements expressing the realism of the game were also generally disagreed with in the Likert-Scale questionnaire, suggesting most participants had some issues with the realism of the simulated experience. Our pursuit of realism during the game development process was heavily motivated by early interviews with farmers, who were the intended audience of the game (Supplementary material 1). Although our sample size of potential users was small and may not be reflective of all the potential users of such games, there was a consistent feeling among interviewees that a research/education game of this sort should reflect real-life scenarios as accurately as possible. However, the difficulties we faced in achieving this desired level of “realism” probably limited the game's potential as a tool for training or assessing farmers' lameness recognition skills. Certainly, some level of realism was achieved; the high accuracy scores of all participants indicated that participants could recognize our virtual lame sheep as lame (Figure 2). However, the lack of an expected difference in recall scores between those with and without farming experience, alongside the lack of an effect of the lameness signs looked for, suggests that our animations were perhaps too obvious. As one farming-experienced participant's feedback attested to, in the field sheep behavior is much more complex (e.g., hiding weaknesses from farmers as part of their prey instinct), and farmers look for a wide variety of body language cues when they observe a sheep's gait for lameness beyond just the textbook examples.

Another possible reason recall scores in the game failed to reflect real-life experience and skills is that the game was not sufficiently engaging for participants to play. Some participants expressed boredom or frustration in the after-game feedback, which is probably the reason many quit the game early (reflected by the wide range of times spent playing in Figure 6). Again, this was partly related to realism; in the pursuit of realism, we probably made the game overly long and sacrificed entertainment value. For example, the decision to program the sheep to only walk intermittently to better reflect real life behavior led us to develop a game that was 10 min long to ensure participants had a sufficient opportunity to observe each of the 25 sheep in the virtual flock walking at least once. Especially considering that the game consisted of repeating one task, this may have caused many participants to quit the game early, impacting their recall scores. Although an overemphasis on the “fun factor” can be detrimental to the use of games in non-gaming contexts like agriculture (50), game-based approaches must still achieve a user experience that is to some extent playful and engaging (51), especially as many people hold preconceived notions that video games are always designed for the purpose of entertainment. More technical problems such as in-game “bugs” and problems participants had engaging with the virtual flock may have further limited the game's engagingness. Again reflecting of the minutiae of signals that farmers process when trying to recognize lameness, in-game malfunctions such as the foot-sliding “bug”—which we considered relatively inconsequential and not a priority (in terms of what was feasible given the predetermined project budget and time frame) to fix before the study roll-out—turned out to be quite distracting for some participants. More generally, the inability to move the virtual sheep and “work the flock” was frustrating for some participants, who expressed that passive observation was not an efficient way to identify lameness.

Finally, we would like to highlight the importance of budgetary limitations in limiting our ability to achieve the levels of realism and engagingness that participants expected. Although we worked with a skilled game programmer and animator experienced in scientific animation, we were not always able to make the most of their skills due to the constraints of our £5,000 budget (Supplementary material 4). This limited the time the game programmer and animator had available to work on the project, and they were thus not always able to make use of the feedback and support that was available from the review and testing stages (e.g., addressing boredom issues or the “foot slide” bug). Furthermore, funding was not sufficient to enable us to hire someone with subject-specific expertise (e.g., a sheep farmer) to directly work with the game developer and animator on a day-to-day basis (which they expressed would have helped). We therefore strongly recommend that future grant applications for serious game projects seek sufficient funding to cover more of the primary game developers' time and also facilitate much closer, more direct collaboration between the game developers and the game's intended audience. This would enable design choices to be driven by the intended audience's involvement and not by what is feasible due to budget limitations, increasing game acceptance and the potential benefits of this medium.

### Insights on lameness recognition practices

Our study did reveal some interesting insights on lameness recognition and produce some evidence of future potential for using games as a tool in livestock husbandry education and research.

Firstly, our inter-disciplinary study points to the way in which animal ailments like lameness may resist precise scientific definitions. Despite the highly controlled *in silico* laboratory we created in which lameness is precisely programmed into the virtual flock, we nonetheless observed a wide variety of recall scores. Although we primarily attribute this to the effect of time spent playing (supported by our quantitative analysis) and the difficulty of adequately mimicking real-life in a video game (supported by our qualitative analysis), our results are also likely to reflect the inherent subjectivity involved in assessing lameness. Previous research has shown that even when observing (videos of) real sheep, farmers and other specialists vary substantially in what they define as lame (especially for early lameness), with different “thresholds” for defining lameness and acting upon it (27). Thus, whilst “*most sheep farmers know the signs of lameness*,” as one participant commented, lameness is a spectrum that may resist a precise definition and be tied up with individual farmers' lived experience. The use of mixed methods reveals this acutely, lending a unique level of support to the hypothesis that subjective experience must be better considered when seeking to design interventions for livestock husbandry issues like lameness in farming.

Similarly, some of our results suggest that the game produced a level of understanding that would not have been so easily achieved with solely survey-based methods, allowing farmers to engage with researchers in novel ways. In particular, we note that the process of researchers illustrating (through the creation of a game) their “vision” of what lameness recognition on the farm looks like (and requesting feedback from those with real-life farming experience on this) facilitated conversations about lameness that perhaps may not have happened with solely survey-based methods—one of the main benefits of the human-centered design approach. Participants reacted strongly to the artificial, simplified world we created, telling us what was missing from our vision and highlighting the limitations of our understanding as academics, proving the utility of iterative prototyping (52). A notable example of this was those with real-life farming experience questioning our assumption that early lameness recognition depended on passive observation and making clear that it depends on actively “working the flock.” Similarly, participant feedback and performance data revealed how academics might misdiagnose real-life problems (and by implication, prescribe flawed solutions); revealing that the decision-making challenge in lameness management may not lie in being able to recognize lameness early, but in being able to act upon this knowledge accordingly (e.g., in finding time and resources to catch and treat sheep). Such assumptions may not have been obvious in a less creative, interdisciplinary project, and has implications for managing lameness in the real-world; suggesting that finding ways to embed lameness reflection and monitoring into existing shepherding practices might help reduce lameness more than trying teach farmers the signs of lameness.

Finally, on a more fundamental level, the game-based, incentivized study appeared to function well as a “hook” to encourage agriculturalists to discuss and participate in a more conventional survey about managing animal health. Many participants shared positive feedback on the game, especially with regards to its potential as an educational tool (even if this had not been fully realized). Furthermore, anecdotally at least, some agriculturalists suggested that the novelty of using a game made the study more appealing (especially when compared to solely survey-based studies that they often get requests to participate in). The game also supported experiential learning through reflection and facilitated the acquisition of up-to-date information on lameness recognition in UK farmers. Agriculturalists were clearly at least trying to spot lameness in the virtual sheep as they would for real-life sheep, and some explicitly expressed that it allowed them to take stock of their real-life practice. The fact that those with farming experience tended to look for lameness signs more often (Supplementary Figure 4) is consistent with the previously reported finding that most farmers know how to identify lameness (27), though a larger sample size would be needed to confirm this. New sociological tools like games may therefore at least help facilitate survey methods and encourage more active participation and engagement between farmers and researchers, as well as support learning through reflection.

### Implications for use of games in livestock husbandry

Our findings have important implications for the future development of games intended as tools to engage with farmers on livestock husbandry issues such as lameness and stockpersonship. In particular, they highlight that future similar projects should consider carefully whether games are best used as “virtual laboratories” to study and train participants, or more as tools to facilitate discussion between researchers and stakeholders in livestock husbandry.

If the games being developed and/or used are intended to be used as “virtual laboratories,” researchers should consider carefully whether the levels of both realism and engagingness that we expect farming audiences to demand of this medium are achievable before initiating the project. A bigger budget, resources, experience and closer engagement with farmers, stockpeople and farm vets will certainly help to tap the full potential of games in this context—though balancing realism and engagement is still likely to be a challenge when using this medium with this audience. One approach that might prove a particularly fruitful avenue for exploration in this regard is to build on existing games, rather than creating games anew. This was the ethos of a recent study that demonstrated the educational value of games in learning natural history by using the professionally-developed video game Red Dead Redemption 2, leveraging its established popularity, realism and entertainment value to engage participants whilst saving time and resources (53). The hyper-real popular video game Farming Simulator—which is already played by farmers (17)—might serve a similar role in future studies of games in agriculture. Indeed, Pavlenko et al. (23) have already had some success building a “mod” (a “modification”—new game content/software created by someone other than the primary game development team) for this game to encourage the adoption of precision agriculture technologies. Alternatively, future projects might do better to use real-life imagery rather than 2/3D models to simulate agricultural environments; this ethos is already being successfully deployed by the “3D farms” project centered around virtual reality to overcome logistically and accessibility challenges in agricultural training (18).

If games are intended to be used more broadly as tools to facilitate discussion between researchers and stakeholders, researchers should be less tied to realism and be more open to letting the game develop organically in close consultation with stakeholders. The game development process itself may facilitate knowledge exchange more than end product, as evidenced by the insights on real-life lameness recognition practices gained through participants telling us what our game was missing, for example. This is something that should be explored in a more dedicated way in future studies using games to engage with farmers on aspects of livestock husbandry.

## Conclusions

The use of games in agricultural research has been increasing in recent years and here, we attempted to develop and use a game to support the study of lameness recognition in UK sheep farmers. We found that besides the positive effects of the game in supporting understanding, knowledge exchange and reflection of lameness, difficulties engaging the agricultural audience limited the potential of the game for education and research. In particular, experienced livestock farmers, stockpeople, and veterinarians requested much higher levels of realism and engagingness than could be achieved with the limited project budget and time-frame.

These results suggest that more needs to be done to establish whether games can be a cost-effective tool in livestock health education and research, and to explore the most effective ways and scenarios in which to use them. Future similar studies should seek to obtain larger budgets, build on existing agricultural simulation games, and work more directly with their target audience, in order to develop games that can more acutely address the challenges of managing livestock health in the twenty-first century.

## Data availability statement

The raw data for this study is deposited at Open Science Framework (https://osf.io/a6qu4/). This data can be used alongside the R/R Markdown code deposited at the lead author's GitHub repository for this study (https://github.com/befriendabacterium/lamenessgame) to reproduce the quantitative analysis of participant recall scores in the game, and the manuscript itself. All data and code (including its outputs) are archived at Zenodo (https://doi.org/10.5281/zenodo.7605244). A copy of the game used in the study is also archived in a separate Zenodo repository (https://doi.org/10.5281/zenodo.7612059), which can be downloaded to play the game locally/offline.

## Ethics statement

The studies involving human participants were reviewed and approved by the School of Informatics Ethics Committee (Cardiff University; human-centered design process) and the College of Medicine and Health Research Ethics Committee (University of Exeter; online evaluation study). The participants provided their informed consent to participate in this study and publish their anonymous data publicly.

## Author contributions

MLJ, MSB, RRH, AKM, and NV were the core team of the project—conceiving the original idea, securing funding *via* writing a grant proposal, designing and conducting the research, and contributing to the writing of the manuscript. MLJ was the project manager, conducting day-to-day management of the project as well as its finances and hires (OM and TL). NV was the secondary project manager, guiding the human-centered design, evaluation process, and supervising OM during his Master's work that formed part of this study (Phases 1 and 2). OM was the game programmer throughout. TL was the 3D artist and animator hired to help produce the final prototype of the game. HMV, MLE, and LG provided expert consultation throughout the project and/or were members of the advisory panel who contributed substantially to the development of sheep models and animations by providing guidance and reference material. All authors provided feedback on the final manuscript. All authors contributed to the article and approved the submitted version.
