# Supplementary material for: Exploring the potential of using simulation games for engaging with sheep farmers about lameness recognition
Source: Front Vet Sci. 2023 Feb 23;10:1079948. doi: 10.3389/fvets.2023.1079948 (PMC9995830; doi:10.3389/fvets.2023.1079948)
Supplement: Supplementary file 1 [file Data_Sheet_1.docx]

# Supplementary Material

**Supplementary Material** **1: Development of the Game**

The game was developed using a human-centered design process that began in June 2020, when our interdisciplinary group of researchers (with expertise in microbiology, engineering, social science, and human-computer interaction) started a small project (with a budget of £5000) to initially explore the potential use of game-based approaches in the context of antibiotic use practices in livestock production (Jones *et al.*, 2020). There were three main phases of development.

*Phase 1: Gathering initial requirements and responses to early ideas about a game about antibiotic use in livestock production*

During the initial requirement gathering stage, we conducted interviews with 3 farmers (2 cattle/sheep, 1 pig) and 1 farm vet recruited through the JustFarmers platform (Jones, 2022). Interviews were ethically approved by Cardiff University School of Informatics Ethics Committee, and comprised two parts - a first part of the interview attempting to understand the participant’s experiences and challenges managing disease in livestock production including the current use of antibiotic in livestock production, and a second part in which we started to explore the design space using iterative prototyping to visualise and communicate two early prototypes (Supplementary Figure [1](#designprocessfig)A) to explore their possibilities, and limitations (Lim, Stolterman and Tenenberg, 2008), as well as to provoke discussions and look for alternative ideas (Fallman, 2008). The first design exploration was a wireframe of a simple game intended to communicate the balance between disease prevention and antibiotic stewardship on the farm (prototype 1a). The second design exploration was a slightly higher fidelity wireframe of a game in which players have to judge which animals to treat with antibiotics by selecting animals and assessing their list of symptoms provided as a journal entry (prototype 1b). These wireframes were demonstrated to the 4 interviewees in online meetings, in order to broadly introduce the concept of a game centered around appropriately treating sick animals with antibiotics; to stimulate discussion with farmers about whether such games might be useful for education (farmers were shown the wireframes via screenshare and asked for their thoughts); and to act as conversation-starters. Participants discussed the early prototypes and provided their feedback on their utility.

The interviews and feedback sessions around the prototypes with farmers and vets were recorded and thematically analyzed to identify major themes and ideas to inform the game’s development (Braun and Clarke, 2006). One of the major findings was that farmers felt that in seeking to make a game to support the reduction of antibiotic use in livestock farming, an explicit emphasis on antibiotic use practices was not necessary. Interviews consistently indicated that antibiotic use practices in livestock production are underpinned by stockpersonship in animal health management and the farmer’s challenges and ability to early recognize animal behavioral signs and physical characteristics of sick animals. Regarding the first version of the prototype, participants highlighted the importance of the realism of the game in relation to the natural surroundings of the farm and the animals. In addition, participants highlighted how unrealistic visual elements (cartoonish looking; Supplementary Figure [1](#designprocessfig)) of the prototype can be distracting. Overall, participants suggested that a fruitful avenue to pursue would be to develop game with a realistic-feel that served as a stockpersonship training tool, expressing the sentiment that being able to spot disease early was more critical and challenging than knowing how to treat it.

*Phase 2: Development of first playable prototype (Where’s Woolly?)*

Building on the findings of the first phase, in the second phase we focused on developing a more higher-fidelity prototype game focusing on stockpersonship within a sheep farming context (given that the stakeholders we were engaging had shared experience working with sheep). The game was loosely intended to support antibiotic stewardship in agriculture by providing an environment for testing, honing and studying farmers’ ability to recognise the early signs of ill health in their livestock - though we remained open to other potential uses of the game throughout our evaluation process.

The prototype we developed (prototype 2) was a game in which players were presented with three scenarios of identifying sick sheep in a flock e.g. identifying animals that were walking slower than other animals, or standing apart from the flock, or not eating (Supplementary Figure [1](#designprocessfig)B). Scenarios were helpful to illustrate the potential and future use of the game as well as to gather feedback and identify potential problems (Bødker, 2000). To add more realism to the game, this prototype was created using the Unity development platform (Unity Technologies, 2015) that facilitated the creation of a 3D virtual environment containing more details such as a grassy terrain, bushes, trees, and more realistic models of the animals (in this case we used an existing sheep model (‘asset’) considering the previous feedback). Seven participants including a sheep/cattle farmer, a veterinary microbiologist, 2 healthcare academics from our networks and 3 of our own team members were asked to play and provide feedback via a Likert Scale questionnaire to explore initial playability of the game, adapted from the MEEGA+ method (Petri, Gresse von Wangenheim and Borgatto, 2017), and a short usability questionnaire focussed on identifying in-game bugs and gathering technical suggestions for improvements. Healthcare academics from our networks and our own team members obviously could not offer a perspective on the game based on real-life farming experience and were more subject to bias in their evaluation, which probably limited this evaluation. However, given the goal of understanding the potential playability of the game and identifying technical issues and fixes, this was less of a concern at this stage.

No formal qualitative analysis was conducted on these data due to the brevity of the information provided. Briefly though, participants provided positive comments about the game’s potential for training and research but also highlighted the need for the game to be more realistic. For example, participants suggested that we seek to include in the game more sheep showing subtle symptoms and provide feedback on whether the animal was treated correctly. Participants also suggested that we include an introduction screen to explain the different roles and game actions to the players as well as different camera angles. Overall, participants recognised the game’s potential to improve livestock health management, especially as an educational tool for inexperienced farmers.

*Phase 3: Development of final prototype (The Lameness game)*

Building on the results of Phases 1 and 2, we chose to develop a game focused on lameness recognition in sheep farmers (Supplementary Figure [1](#designprocessfig)C). Lameness was chosen as the theme of the final prototype not only because it provided a focal point for developing a more realistic game, but because it was closely intertwined with stockpersonship, resonated with many of the (mainly sheep and cattle) farmers and vets we consulted, and is a key challenge in UK livestock farming with wide-ranging implications for productivity, welfare and antibiotic stewardship.

For the development of the final prototype that was evaluated in this study, we first enrolled an animator (TL) with experience with scientific animation to work with our game programmer (OM), focusing on developing a realistic animation of lame and non-lame sheep which could form the basis of a game to test farmers’ lameness recognition skills. This was done through a mix of consulting scientific source materials, written and video, mainly from Kaler, Wassink and Green (2009), scientific experts and producing our own reference material (co-author HV filming her own sheep). This information was used to modify an existing 3D sheep model and its animations purchased from the Unity Assets store (Bilalov, 2020), which was then integrated into the game. We created an expert advisory panel of farmers and sheep lameness academics, including some of the co-authors. The first author conducted a one-hour focus group to consult with stakeholders and receive feedback on the animation, aesthetics, gameplay mechanisms and future refinements. Notes were taken during the consultation sessions with stakeholders, which informed the development of the game (though no formal thematic analysis was conducted due to time and resource constraints). Feedback from the advisory panel emphasized the need to improve the sheep gait animations, which we responded to by investing more time and resources into animation refinement and their smooth integration into the game.

**Supplementary Material** **2: Questionnaire**

**Consent**

1. By checking this box I confirm that I have understood and agree with all of the above

statements and I consent to taking part in this project. You must tick this box to agree

with all of the above statements, in order to part in the questionnaire.

**Game Results**

You will need to record your game time & scores after playing the game so please read the instructions below carefully before playing:

STUDY INSTRUCTIONS:

1. Go to <https://wheres-woolly.itch.io/lameness-game,> leaving this form open

2. Play the tutorial, the afterwards play the Game itself

3. Upon finishing the game DO NOT CLOSE THE WEBPAGE - you will be shown your scores (an example screenshot is shown above) - keep it open and enter your scores in the form below, then continue with the rest of the questionnaire.

REMINDER - GAME RECOMMENDATIONS:

- Desktop or laptop computer - The game should not be played on touchscreen devices (i.e. smartphone or tablet).

- Mouse with a scroll wheel or a laptop trackpad - to ensure efficient game-play.

- We recommend playing the game in one of the following web-browsers: Microsoft Edge, Google Chrome or Mozilla Firefox (other browsers are not supported)

- If the game is running slowly, try closing unused web-browser tabs (not this one)

_____________________________________________________

1. Time remaining on clock when you ended the game by clicking ’Done (in the format nnn seconds e.g. 596 seconds in the example screenshot).
2. Lame sheep identified (%) - e.g. 0 in the example screenshot
3. accuracy (%) - e.g. 0 in the example screenshot
4. How many times did you play the game before getting these results (0 = it was my first time playing)?
5. Did you play the tutorial before playing the game?

- Yes, and observed the sheep walking
- Yes, but didn’t observe the sheep walking
- No

1. What computer hardware did you use to play the game? (select all those appropriate)

- Laptop or Desktop computer
- Mouse with scroll-wheel
- Track-pad with pinch zoom
- Smartphone or tablet
- Other

1. Did you experience any problems using the controls or playing the game?

- Yes
- No

1. If yes, please specify

**Game Strategy**

1. What was your strategy for observing the sheep (tick all that apply)?

- [Data plotted in Figure [5](#UEnulleffects)C]
- Observed the whole flock and then zoomed in when I saw one that looked lame
- [Denoted as ‘Zoomer’ in Figure [5](#UEnulleffects)C]
- Observed each sheep up-close until I could see whether or not it had a sign, then moved onto the next sheep
- [Denoted as ‘Up-close’ in Figure [5](#UEnulleffects)C]
- Other (please provide details below)
- [Denoted as ‘Other’ in Figure [5](#UEnulleffects)C]

1. Please provide brief details about your strategy for observing the sheep?
2. How did you move from sheep to sheep (tick one)?

- [Data plotted in Figure [5](#UEnulleffects)D]
- Randomly
- [Denoted as ‘Randomly’ in Figure [5](#UEnulleffects)D]
- Semi-randomly
- [Denoted as ‘Semi-randomly’ in Figure [5](#UEnulleffects)D]
- Started at one end of the flock and worked my way to the other
- [Denoted as ‘Linear’ in Figure [5](#UEnulleffects)D]
- Other (please provide details below)
- [Denoted as ‘Other’ in Figure [5](#UEnulleffects)D]

1. Please provide brief details about how you moved from sheep to sheep
2. What signs did you look for to find the lame sheep (tick all that apply)

- Uneven posture
- Shortened stride on one leg when walking
- Pair of legs which were moving at different speeds
- Nodding of head
- Not weight bearing on affected leg when standing
- Not weight bearing on affected leg when walking
- Reluctance to move
- Slower walking pace
- Other

1. If you answered ‘Other’, please provide brief details about what signs you looked for to find lame sheep

**Real-world experience**

1. Have you ever worked in farming or a related field (e.g. farm vet)?

- Yes
- No

1. How many years have you worked with sheep?
2. In what roles, if any, did you work with sheep (e.g. farmer, stockman/woman/person, veterinarian)?

- Farmer
- Stockman/woman/person
- Veterinarian
- Other

1. If you answered ‘Other’, please provide some brief details about the role(s) in which you worked with sheep
2. What do you think was the average level of lameness in the flock(s) with which you worked/work, over one year?

- Under 2%
- Between 2 and 5%
- Between 5 and 10%
- Over 10%

**Game Feedback**

Please fill in the table below with an indicating how strongly you agree with the preceding statement with 5 being strongly agree and 1 being strongly disagree

How strongly do you agree with the following statements?

1. The game is a realistic representation of recognising sheep lameness in the field
2. Learning to play this game was easy
3. The game rules are clear and easy to understand
4. The contents and structure helped me to become confident that I would learn with this game
5. This game is appropriately challenging for me
6. The game does not become monotonous as it progresses
7. I am motivated to achieve a better score
8. Completing the game tasks gave me a satisfying feeling of accomplishment
9. It is due to my personal effort that I managed to advance in the game
10. I feel satisfied with the things that I learned from the game
11. I would recommend this game to my colleagues/friends
12. I had fun with the game
13. I would play this game again
14. I would recommend this game as a form of entertainment
15. I achieved the goals of the game applying my knowledge
16. I would recommend this game as a form of training/educational tool
17. I was so involved in my gaming task that I lost track of time
18. I forgot about my immediate surroundings while playing this game
19. The game contents are relevant to my interests
20. It is clear to me how the contents of the game are related to my profession

**Supplementary Material** **3: Thematic analysis results**

**List of comments for qualitative analysis (note the typing errors from the original, should mark as [cic] if quoting in text)**

*N.B. Participant numbers do not go from 1 to 63 because there were a few rounds of testing the questionnaire before study roll-out, which affected the participant counter. The participant counter for the actual study therefore starts at 8 and ends at 71 (64 participants; note the participant 36 was removed from the study because their comments indicated the game did not work properly for them, hence the final sample size was 63). Only participants with real-life farming experience were invited to provide feedback on the game and even then this was optional, hence not all participants 8-71 are featured in the below quotes.*

Participant 8: If there was a way to make each sheep move, that would really help to keep engagement, I got bored waiting for the sheep to move unfortunately.

Participant 15: :) [Happy face]

Participant 17: I think most farmers would ay that they also assess lameness by making the sheep walk / move away from them rather than just wait until they walk.

Participant 18: If you wanted to complete the game in a shorter time, you would want the sheep to move around more. I got bored waiting for them to walk. Needs a dog to run round them!

Participant 19: Game animations were not smooth, making the distinction between a normal walking gait and a limp less easy to discern. This scenario may not be very representative, as in my experience lameness is not often identified when animals are static in the field, more often when animals are being moved or handled.

Participant 22: Lame sheep aren’t always that easy to spot in a field

Participant 24: Would be good to get sheep to move, maybe by walking a person around so they walk away from you as it is difficult to assess them systematically. .

Participant 27: i got annoyed waiting for the sheep to move. in a flock i would walk around them and the sheep would move.

Participant 34: The graphics werent very clear - it was hard to see if they were holding a leg slightly up. In reality you would move the sheep to look for lameness

Participant 35: I would have enjoyed this game better if the controls worked better . the sheep animations are good, but to a trained eye i found them confusing , eg none of them stood grazing in a normal posture because they were all jiggling their legs all the time

Participant 38: none [Cannot include in the analysis]

Participant 43: it was entertaiing but i felt there could be improvements made as you chose the right animals maybe a sound so you know your going the right way or a counter in the corner

Participant 44: took a long while for the seep to start moving in the tutorial that i wondered if it was going to move, but I think that’s the point of the questions asking about if I watched the sheep move. i enjoyed the game as it allowed me to get a better sense of my knowledge and skills. it mimicked sheep well but was sometimes difficult to tell if a normal movement of sheep we a game lag.

Participant 50: I thought lameness was really realistic- but was expecting more variation (ie from very early to very severe, different legs, etc - though maybe I didn’t spot that!)

Participant 51: Found it very frustrating. Not realistic. Movement stilted which made identifying slightly lame sheep virtually impossible. Most of the time all sheep standing still, leading to frustration with the game and rushing.

Participant 57: I thought this was brilliant. It was a bit frustrating not to be able to mark non-lame sheep when surveying, but that is more realistic and requires strategy. The main issue was the unrealistic movement of the feet on the ground whilst standing. On my PC there was a foot slide effect. I didn’t look for standing signs as I thought they were more graphics errors

Participant 67: Could be enhanced by slightly more realistic depiction of sheep movement for non-lame sheep

Participant 68: It’s interesting to be looking for sign in virtual sheep, but I got frustrated that I was not able to make them move as would be the case in real life.

Participant 70: very basic. would be nice to have a method of encouraging sheep to move. In real life I would walk around the flock and observe they way the moved. In this game the sheep were fairly stationary which made that hard.

**First reviewer’s (M.S.B) comments on second reviewer’s (N.V.D) analysis and points of difference**

The two analyses presented are compatible to a large extent and reflect far more commonalities than fundamental points of difference. Where there were discrepancies, these reflected different professional backgrounds and differential prioritisation of aspects of the dataset, especially relating to technical versus experiential aspects.

M.S.B. identified 4 themes:

1.       Challenges of identifying lameness

2.       Psychological responses

3.       Realism of farming simulation

4.       Technical performance

N.V.D. identified 5 themes

1.       Perceived Realism of the Game

2.       Reflective experiences

3.       Challenges of the Game simulation

4.       Emotional Responses to the Game

5.       Participant’s suggestions for improvements

I consider that N.V.D has captured the content of my themes, with the following comments.

M.S.B. opted to sort the themes alphabetically. N.V.D. has not stated a logic for ordering the themes. I would prefer to retain alphabetical ordering (unless a strong rationale to the contrary is provided).

I would prefer to retain the theme title ‘Psychological responses’ rather than ‘Emotional responses’, but am happy to add ‘to the game’. I consider that the term ‘Psychological’ better captures the range of sub-themes.

I consider the only amendments needed to N.V.D.’s coding are to order alphabetically and to replace ‘emotional’ by ‘psychological’

**Results/themes identified**

1.       Perceived realism of the game (PR)

o   Quote 1: “the sheep animations are good” (Participant 35)

o   Quote 2: “it mimicked sheep well” (Participant 44)

o   Quote 3: “I thought lameness was really realistic…” (Participant 50)

o   Quote 4: “Not realistic.” (Participant 51)

o   Quote 5: “The main issue was the unrealistic movement of the feet on the ground whilst standing” (Participant 57)

2.       Technical challenges playing the simulation game (TC)

·       Sub-theme 1: Lack of movement of the sheep

o   Quote 1: “: I think most farmers would say that they also assess lameness by making te sheep walk / move away from them rather than just wait until they walk” (Participant 17)

o   Quote 2: “as in my experience lameness is not often identified when animals are static in the field, more often when animals are being moved or handled.” (Participant 19)

o   Quote 3: “I got annoyed waiting for the sheep to move. in a flock i would walk around them and the sheep would move.” (Participant 27)

o   Quote 4: “In reality you would move the sheep to look for lameness” (Participant 34)

o   Quote 5: “took a long while for the sheep to start moving in the tutorial that i wondered if it was going to move, but I think that’s the point of the questions asking about if I watched the sheep move” (Participant 44)

o   Quote 6: “Most of the time all sheep standing still, leading to frustration with the game and rushing” (Participant 51)

o   Quote 7: “I was not able to make them move as would be the case in real life.” (Participant 68)

·       Sub-theme 2: Simple, unnatural, and confusing game simulation of sheep’s behaviour (SB)

o   Quote 1: “the sheep animations are good, but to a trained eye i found them confusing , eg none of them stood grazing in a normal posture because they were all jiggling their legs all the time.” (Participant 35)

o   Quote 2: “was sometimes difficult to tell if a normal movement of sheep we a game lag.” (Participant 44)

o   Quote 3: “was expecting more variation (ie from very early to very severe, different legs, etc - though maybe I didn’t spot that!)” (Participant 50)

o   Quote 4: “Movement stilted which made identifying slightly lame sheep virtually impossible.” (Participant 51)

o   Quote 5: “Very basic… In this game the sheep were fairly stationary which made that hard” (Participant 70)

·       Sub-theme 3: Unable to mark non-lame sheep

o   Quote 1: “It was a bit frustrating not to be able to mark non-lame sheep when surveying, but that is more realistic and requires strategy.” (Participant 57)

·       Sub-theme 4: Usability and Animation/simulation issues (e.g., transitions, controls, graphics) (UA)

o   Quote 1: “Game animations were not smooth, making the distinction between a normal walking gait and a limp less easy to discern.” (Participant 19)

o   Quote 2: “The graphics werent very clear - it was hard to see if they were holding a leg slightly up.” (Participant 34)

o   Quote 3: “would have enjoyed this game better if the this game if the controls worked better” (Participant 35)

o   Quote 4: “… On my PC there was a foot slide effect. I didn’t look for standing signs as I thought they were more graphics errors” (Participant 57)

3.       Emotional responses to the game (ER)

·       Sub-theme 1: Enjoyment

o   Quote 1: “:) [Happy face]” (Participant 15)

o   Quote 2: “: it was entertaining” [Sic] (Participant 43)

o   Quote 3: “I enjoyed the game” (Participant 44)

·       Sub-theme 2: Surprise/interesting

o   Quote 1: “I thought this was brilliant” (Participant 57)

o   Quote 2: “It’s interesting to be looking for sign in virtual sheep” (Participant 68)

·       Sub-theme 3: Boredom

o   Quote 1: “I got bored waiting for the sheep to move unfortunately” (Participant 8)

o   Quote 2: “I got bored waiting for them to walk” (Participant 18)

·       Sub-theme 4: Frustration

o   Quote 1: “i got annoyed waiting for the sheep to move” [Sic] (Participant 27)

o   Quote 2: “Found it very frustrating.” (Participant 51)

o   Quote 3: “But I got frustrated.” (Participant 68)

4.       Reflective experiences

o   Quote 1: “Lame sheep aren’t always that easy to spot in a field” (Participant 22)

o   Quote 2: “in a flock i would walk around them and the sheep would move” (Participant 27)

o   Quote 3: “it allowed me to get a better sense of my knowledge and skills” (Participant 44)

o   Quote 4: “In real life I would walk around the flock and observe they way the moved” (Participant 70)

5.       Participants’ suggestions for improvements

·       Sub-theme 1: Making sheep move e.g., using additional mechanisms and characters

o   Quote 1: “If there was a way to make each sheep move, that would really help to keep engagement” (Participant 8)

o   Quote 2: “If you wanted to complete the game in a shorter time, you would want the sheep to move around more… Needs a dog to run round them!” (Participant 18)

o   Quote 3: “Would be good to get sheep to move, maybe by walking a person around so they walk away from you…” (Participant 24)

o   Quote 4: “would be nice to have a method of encouraging sheep to move…” (Participant 70)

·       Sub-theme 2: Providing additional visual/sound feedback

o   Quote 1: “i felt there could be improvements made as you chose the right animals maybe a sound so you know your going the right way or a counter in the corner…” (Participant 43)

o   Quote 2: “Could be enhanced by slightly more realistic depiction of sheep movement for non-lame sheep” (Participant 67[JM1] )

**Supplementary Material** **4: Project Budget Breakdown**

| **Expense** | **Spend (£)** |
| --- | --- |
| Game Developer (hired at a rate £20.55ph broken down into £18.43ph basic rate + £2.21ph holiday pay) | 3,674 |
| 3D artist/animator (hired at a rate £20.55ph) | 935 |
| Digital models from the Unity Asset store that were used in the game | 66 |
| Participant incentivisation (£70 gift vouchers to reimburse/thank early-phase interviewees; £40 for study questionnaire testers, £150 on 3 x £50 Chelford Farm Supplies vouchers as lottery prizes/incentives for participating in the final study) | 260 |
| Promoting the study in the National Sheep Association’s newsletter | 42 |
| TOTAL | 4,978 |

**Supplementary Figure** **1:** Description of the human-centered design process. A) Screenshots of the initial wireframes/prototypes 1a and 1b developed around the concept of antibiotic use in livestock farming; B) Screenshot of prototype 2, 'Where's Woolly' in which players are challenged to identify simple signs of illness in sheep; C) Screenshot of the animations and final game in which they were used, which is the game evaluated in this study.


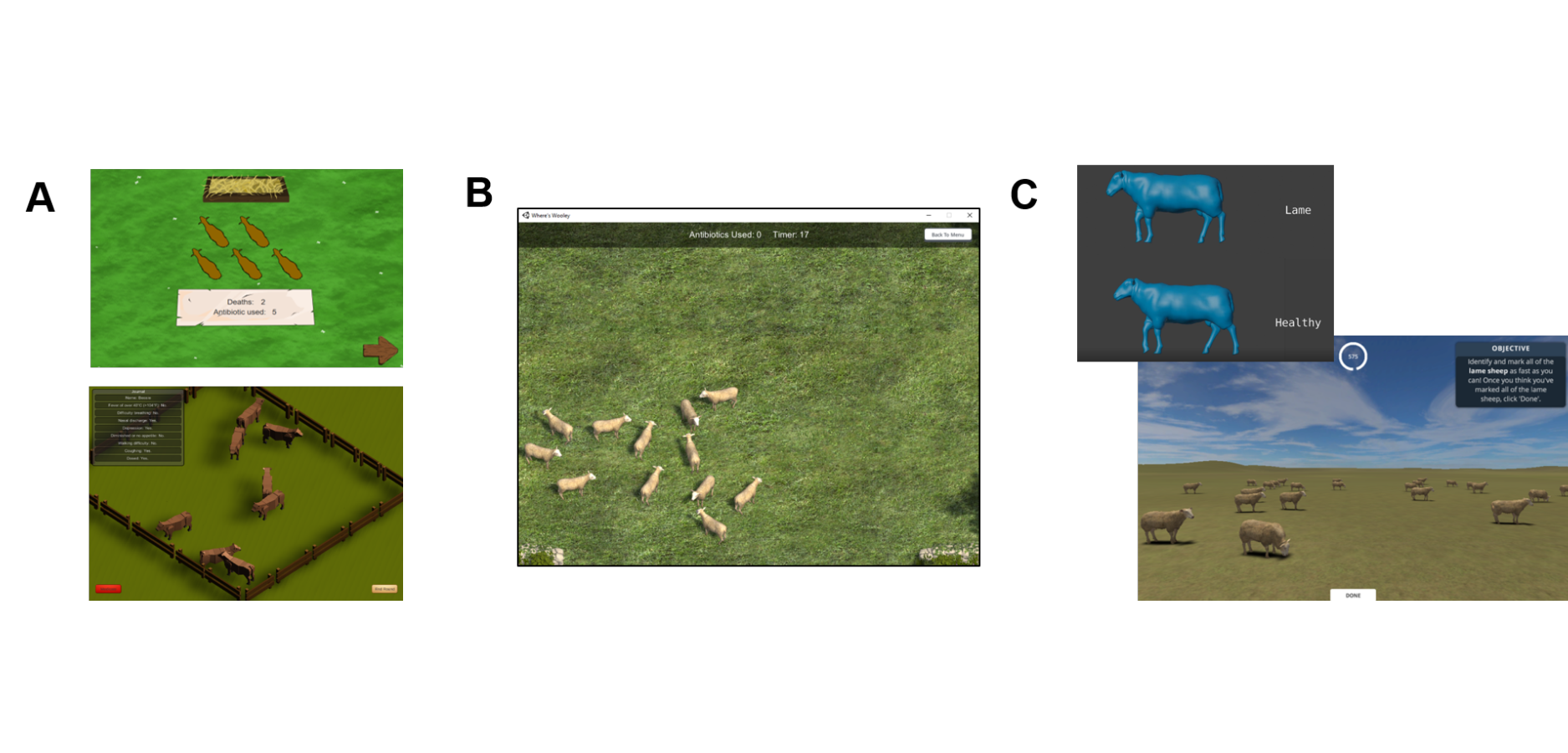


**Supplementary Figure** **2:** Relationships between participants' recall scores and real-life experience working with sheep as a A) Farmer; B) Stockman/woman/person C) Veterinarian D) Other e.g. academic, policy. Individual participant data points are jittered using the beeswarm algorithm (R Package 'beeswarm') and mean recall scores are plotted as bold horizontal lines underneath the data points. Mean recall scores coloured red are those likely to be poor estimates due to small sample sizes i.e. the lower or upper quartile exceeds the 95% confidence limits of the mean. The plot is framed in a bold outline if that relationship was formally tested statistically.


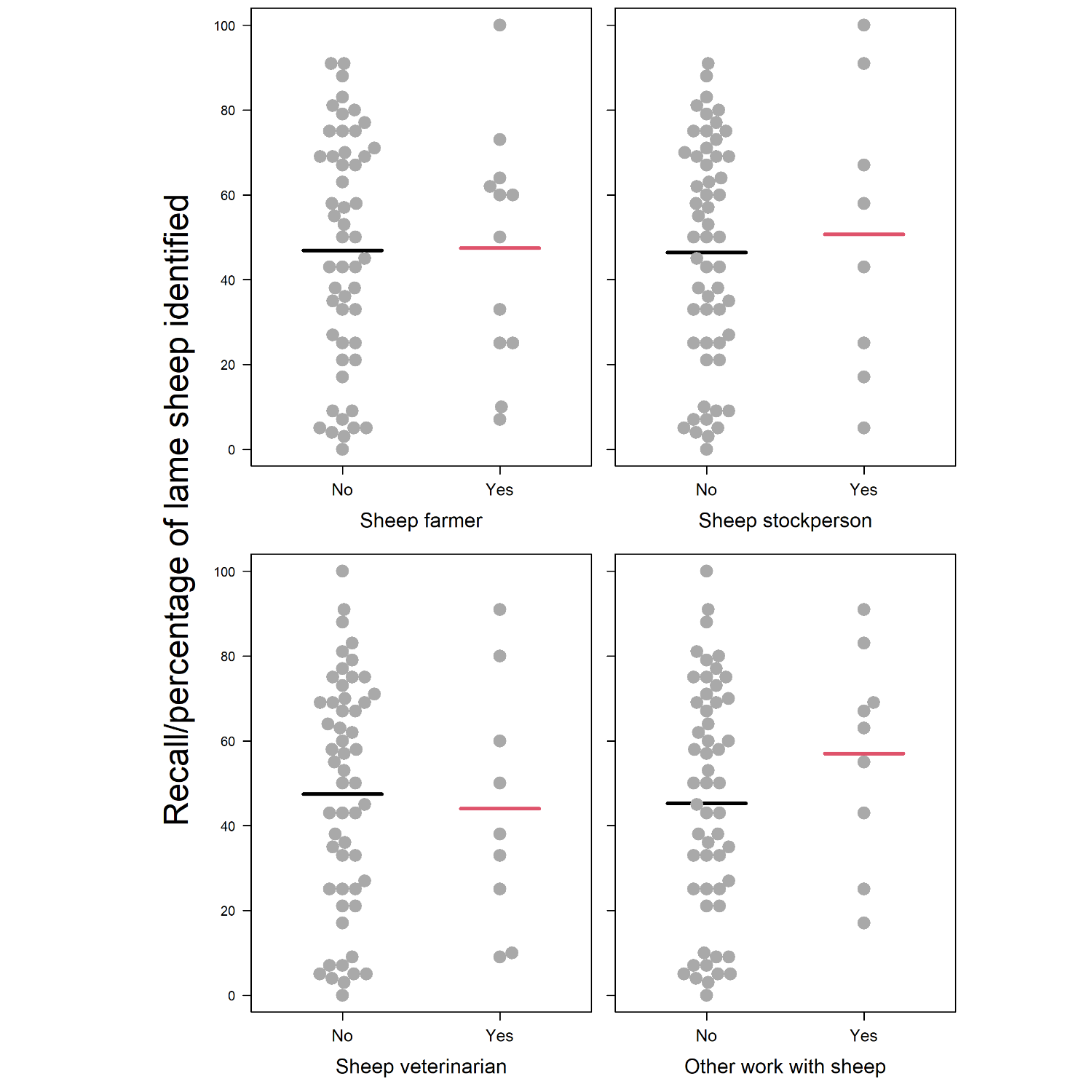


**Supplementary Figure** **3**: Diagnostic plots for the 'Farming Experience' model. From top left to bottom right: Residuals vs Fitted plot showing if residuals have linear patterns (residuals should be approximately equally spread around the horizontal red line if so); Normal Q-Q plot showing if residuals are normally distributed (residuals should approximately follow the dashed line if so); Scale-Location plot showing if residuals are spread equally along the ranges of the predictors (residuals should be approximately equally spread around the horizontal red line if so); Residuals vs Leverage plot to identify any outliers that are influential in the linear regression (Cook's distance lines should not be visible and/or points should all be within Cook's distance lines)


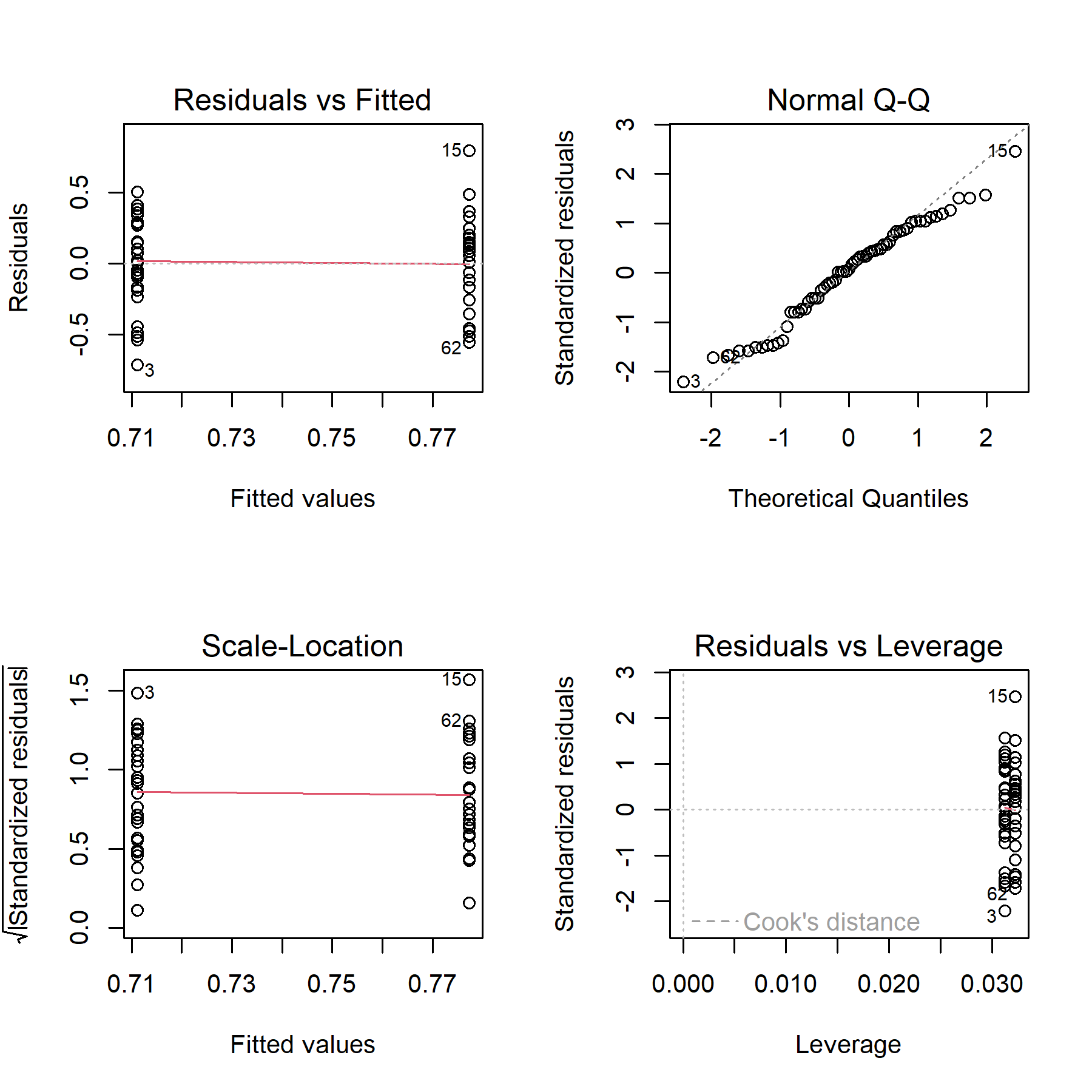


**Supplementary Figure** **4:** Balloon plot of contingency table used to conduct chi-squared test for a difference in the lameness signs looked for according to real-life farming experience. Size of the circles/balloons reflects the frequency of participants that looked for that lameness sign (relative to the total number of signs looked for by both groups)


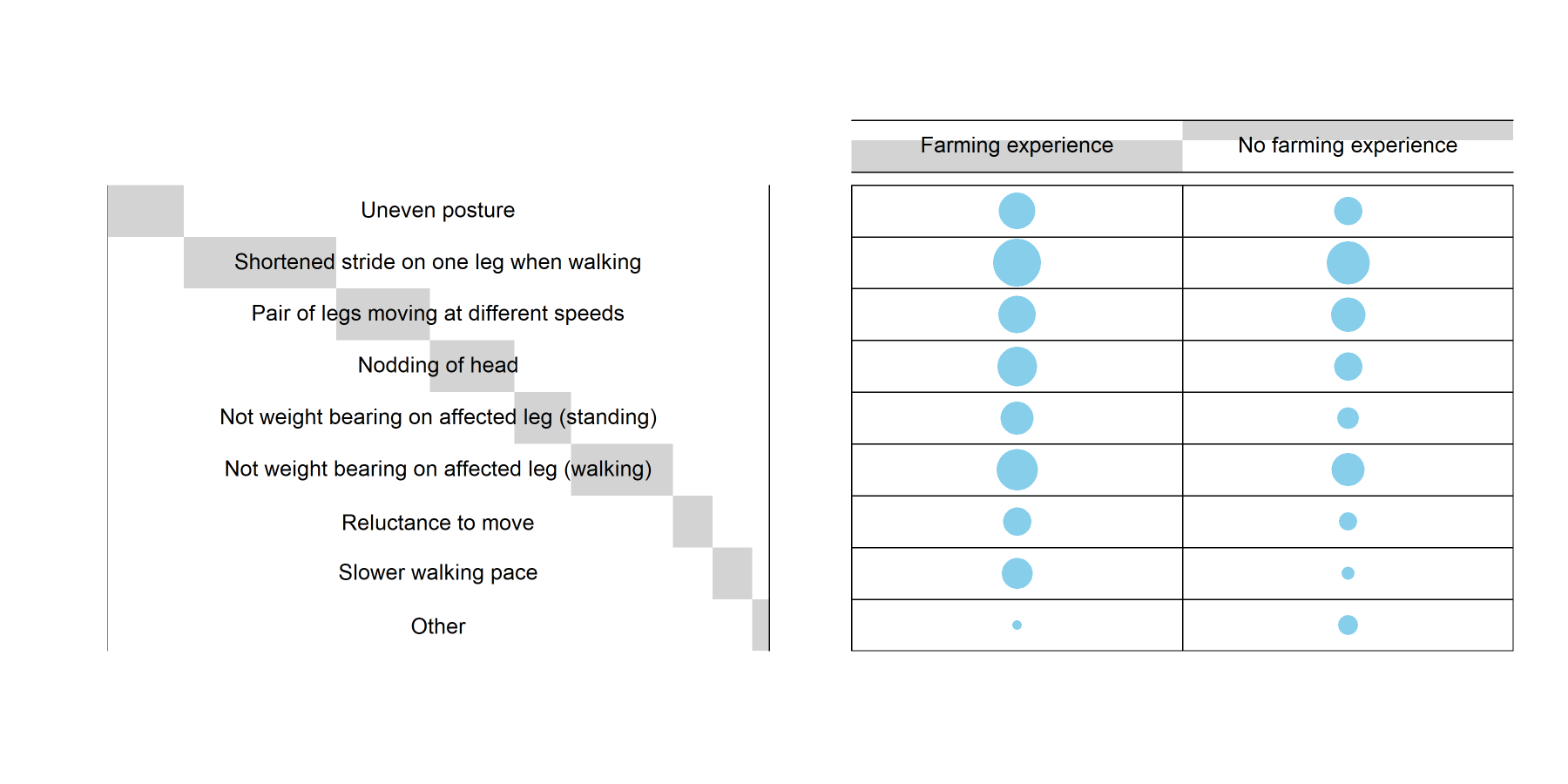


**Supplementary Figure** **5:** Diagnostic plots for the 'Lameness signs looked for' model A (uneven posture) model. From top left to bottom right: Residuals vs Fitted plot showing if residuals have linear patterns (residuals should be approximately equally spread around the horizontal red line if so); Normal Q-Q plot showing if residuals are normally distributed (residuals should approximately follow the dashed line if so); Scale-Location plot showing if residuals are spread equally along the ranges of the predictors (residuals should be approximately equally spread around the horizontal red line if so); Residuals vs Leverage plot to identify any outliers that are influential in the linear regression (Cook's distance lines should not be visible and/or points should all be within Cook's distance lines)


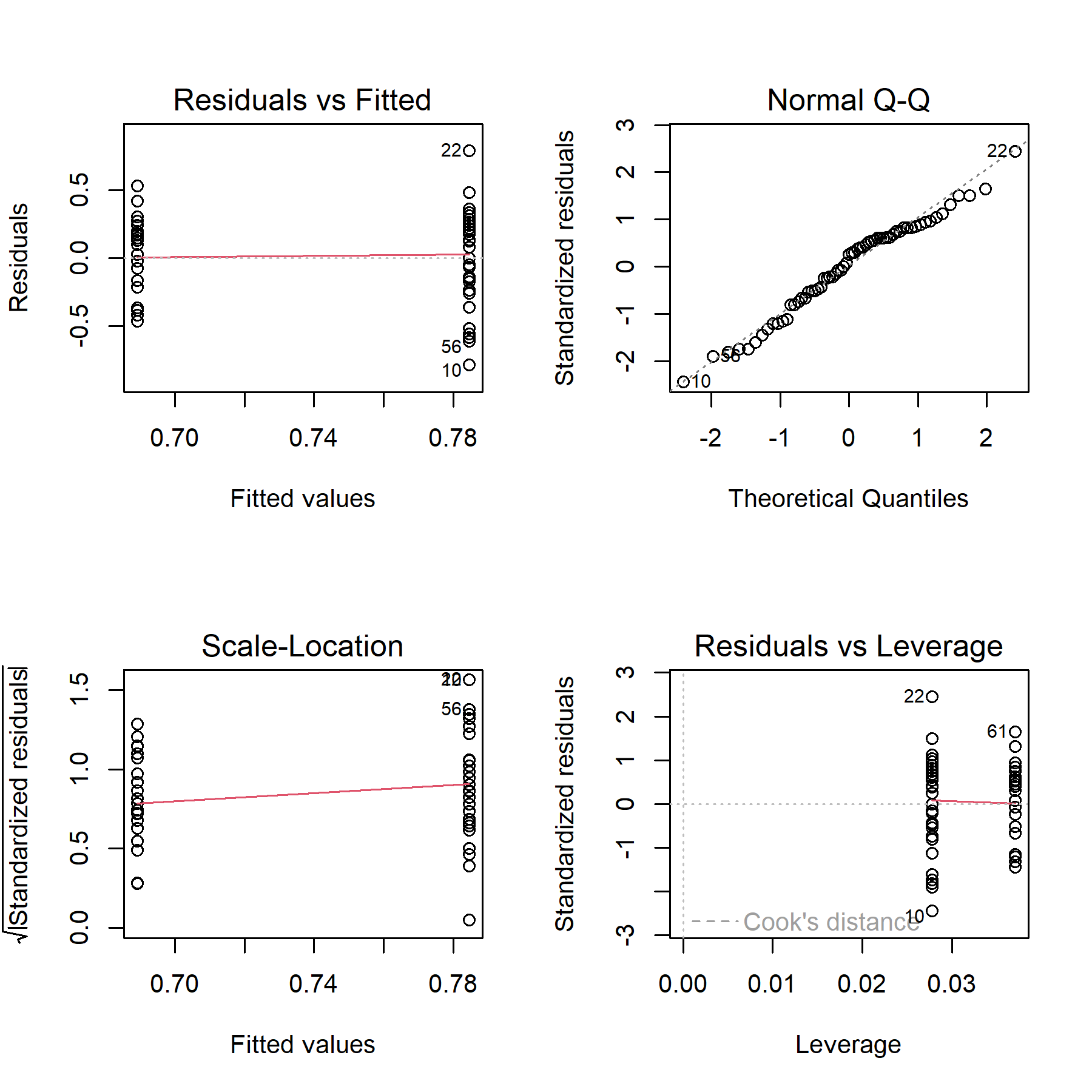


**Supplementary Figure** **6:** Diagnostic plots for the 'Lameness signs looked for' model B (limp) model. From top left to bottom right: Residuals vs Fitted plot showing if residuals have linear patterns (residuals should be approximately equally spread around the horizontal red line if so); Normal Q-Q plot showing if residuals are normally distributed (residuals should approximately follow the dashed line if so); Scale-Location plot showing if residuals are spread equally along the ranges of the predictors (residuals should be approximately equally spread around the horizontal red line if so); Residuals vs Leverage plot to identify any outliers that are influential in the linear regression (Cook's distance lines should not be visible and/or points should all be within Cook's distance lines)


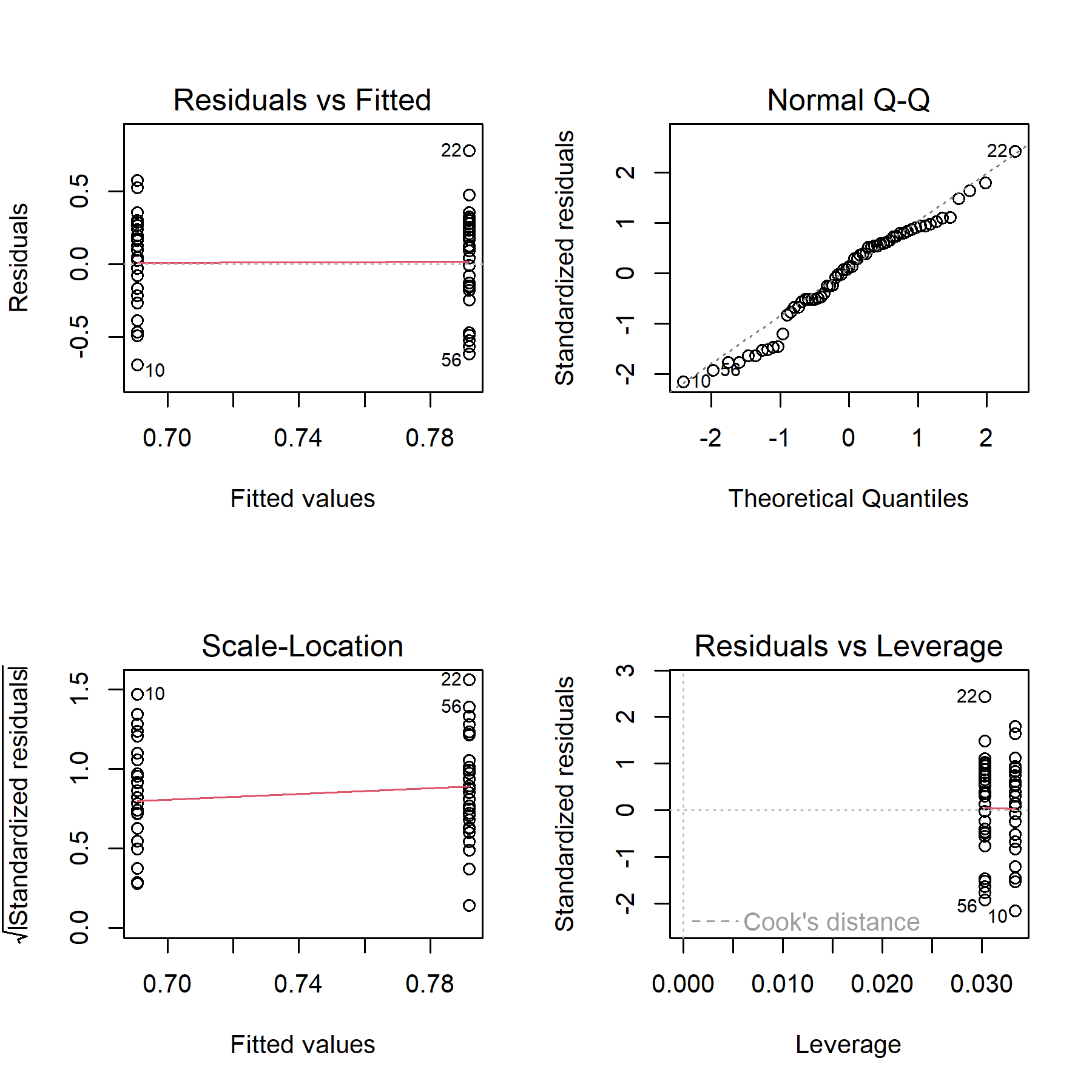


**Supplementary Figure** **7:** Diagnostic plots for the 'Lameness signs looked for' model C (raised leg) model. From top left to bottom right: Residuals vs Fitted plot showing if residuals have linear patterns (residuals should be approximately equally spread around the horizontal red line if so); Normal Q-Q plot showing if residuals are normally distributed (residuals should approximately follow the dashed line if so); Scale-Location plot showing if residuals are spread equally along the ranges of the predictors (residuals should be approximately equally spread around the horizontal red line if so); Residuals vs Leverage plot to identify any outliers that are influential in the linear regression (Cook's distance lines should not be visible and/or points should all be within Cook's distance lines)


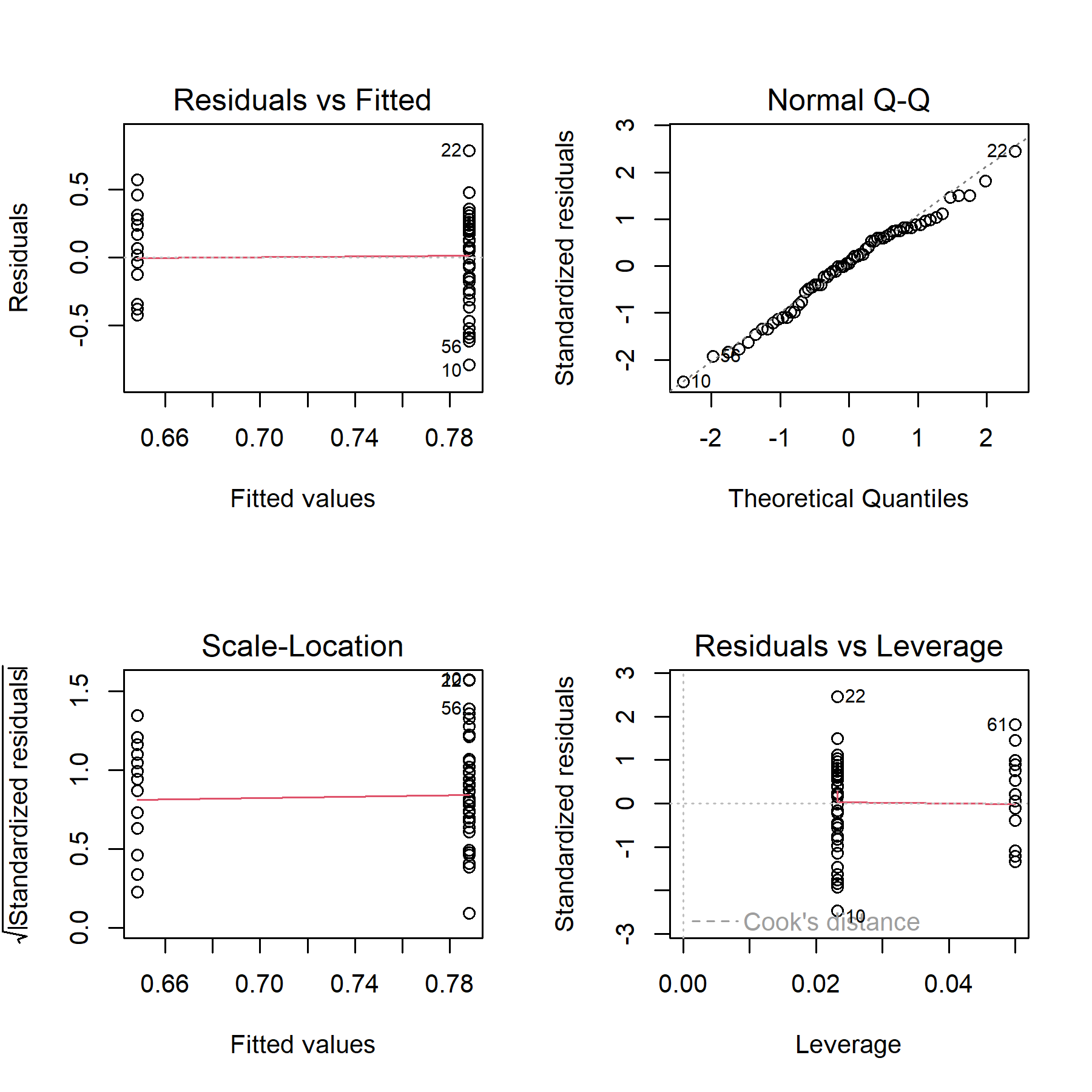


**Supplementary Figure** **8:** Diagnositc plots for the 'User Engagement' model. From top left to bottom right: Residuals vs Fitted plot showing if residuals have linear patterns (residuals should be approximately equally spread around the horizontal red line if so); Normal Q-Q plot showing if residuals are normally distributed (residuals should approximately follow the dashed line if so); Scale-Location plot showing if residuals are spread equally along the ranges of the predictors (residuals should be approximately equally spread around the horizontal red line if so); Residuals vs Leverage plot to identify any outliers that are influential in the linear regression (Cook's distance lines should not be visible and/or points should all be within Cook's distance lines)


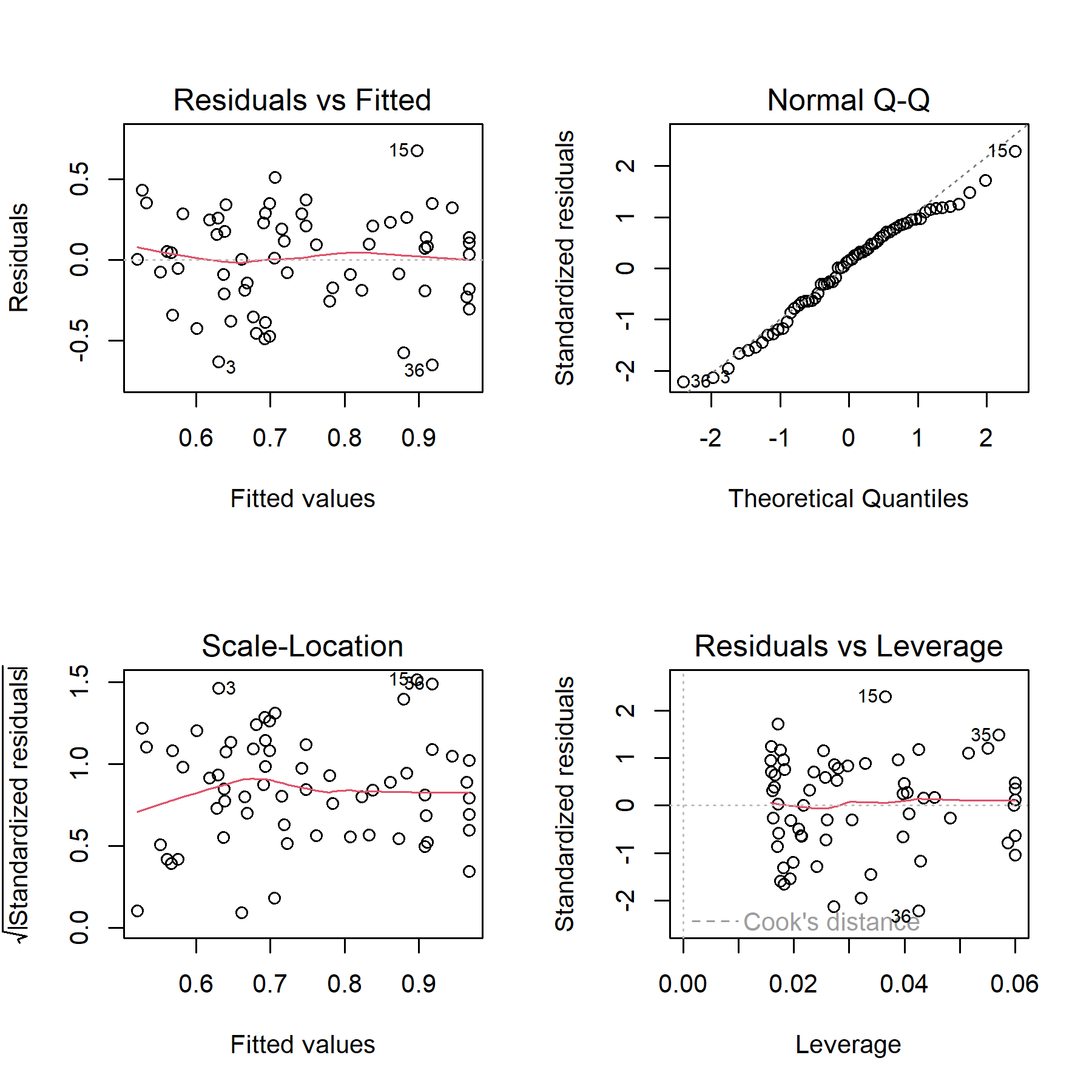


# References (supplementary)

Jones, M., Hughes, R., Murray, A., Verdezoto, N. and Barnish, M. (2020) ‘Exploring antibiotic use practices in livestock production through a novel, game-based approach’. Available at: <https://gw4.ac.uk/exploring-antibiotic-use-practices-in-livestock-production-through-a-novel-game-based-approach/>.

Jones, A. (2022) ‘Just farmers’. Available at: <https://www.justfarmers.org/>.

Lim, Y.-K., Stolterman, E. and Tenenberg, J. (2008) ‘The anatomy of prototypes: Prototypes as filters, prototypes as manifestations of design ideas’, *ACM Transactions on Computer-Human Interaction*, 15(2), pp. 7:1–7:27. Available at: <https://doi.org/10.1145/1375761.1375762>.

Fallman, D. (2008) ‘The Interaction Design Research Triangle of Design Practice, Design Studies, and Design Exploration’, *Design Issues*, 24(3), pp. 4–18. Available at: <https://www.jstor.org/stable/25224179> (Accessed: 31 August 2022).

Braun, V. and Clarke, V. (2006) ‘Using thematic analysis in psychology’, *Qualitative Research in Psychology*, 3(2), pp. 77–101. Available at: <https://doi.org/10.1191/1478088706qp063oa>.

Bødker, S. (2000) ‘Scenarios in user-centred design—setting the stage for reflection and action’, *Interacting with Computers*, 13(1), pp. 61–75. Available at: <https://doi.org/10.1016/S0953-5438(00)00024-2>.

Petri, G., Gresse von Wangenheim, C. and Borgatto, A.F. (2017) ‘MEEGA+, Systematic Model to Evaluate Educational Games’, in N. Lee (ed.). Cham, Switzerland: Springer International Publishing, pp. 1–7. Available at: <https://doi.org/10.1007/978-3-319-08234-9_214-1>.

Unity Technologies (2015) *Unity*. San Francisco, California: Unity Software Inc. Available at: <https://unity.com/>.

Kaler, J., Wassink, G.J. and Green, L. (2009) ‘The inter- and intra-observer reliability of a locomotion scoring scale for sheep’, *The Veterinary Journal*, 180(2), pp. 189–194. Available at: <https://doi.org/10.1016/j.tvjl.2007.12.028>.

Bilalov, R. (2020) *Sheep realistic | characters | unity asset store*. Uralsk, Kazakhstan: Red Deer. Available at: <https://assetstore.unity.com/packages/3d/characters/animals/mammals/sheep-realistic-176904>.
